# ADRB2 polymorphism Arg16Gly modifies the natural outcome of heart failure and dictates therapeutic response to β-blockers in patients with heart failure

**DOI:** 10.1038/s41421-018-0058-6

**Published:** 2018-10-23

**Authors:** Jin Huang, Chenze Li, Ying Song, Xiaohan Fan, Ling You, Lun Tan, Lei Xiao, Qing Li, Guoran Ruan, Senlin Hu, Wei Cui, Zongzhe Li, Li Ni, Chen Chen, Anthony Yiu-Ho Woo, Rui-Ping Xiao, Dao Wen Wang

**Affiliations:** 1 Division of Cardiology, Departments of Internal Medicine, Tongji Hospital, Tongji Medical College, Huazhong University of Science & Technology, 430030 Wuhan, China; 2 Hubei Key Laboratory of Genetics and Molecular Mechanisms of Cardiologic Disorders, 430030 Wuhan, China; 3 Institute of Molecular Medicine, Peking-Tsinghua Centre for Life Sciences, Peking University, 100871 Beijing, China; 4 Fuwai Hospital, Chinese Academy of Medical Sciences and Peking Union Medical College, 100037 Beijing, China; 5 Division of Cardiology, The Second Hospital of Hebei Medical University, 050000 Shijiazhuang, China; 6 Department of Pharmacology, School of Life Sciences and Biopharmaceutics, Shenyang Pharmaceutical University, 110016 Shenyang, China

## Abstract

We sought to investigate the association of single nucleotide polymorphisms (SNPs) of the genes involved in βAR signaling with the response of patients to βAR blockers. A total of 2403 hospitalized patients with chronic heart failure (HF) were enrolled in a multicenter observational study as the first cohort and followed up for a mean period of 20 months. Genes for β1AR, β2AR, and the major cardiac G-protein-coupled receptor kinases (GRKs) *GRK2* and *GRK5* were analyzed to identify SNPs, and patients were stratified according to genotypes. A second independent cohort enrolling 919 patients with chronic HF was applied to validate the observed associations. The signaling properties of the key identified SNPs were assessed in vitro. Our data showed that HF patients harboring the Gly16 allele in the gene for β2AR (*ADRB2*) had an increased risk of the composite end point relative to patients who were homozygous for Arg16. Notably, these patients showed a beneficial response to βAR-blocker treatment in a G allele-dose-dependent manner, whereas Arg16 homozygotes had no response to βAR-blocker therapy. This Arg16Gly genotype-dependent heterogeneity in clinical outcomes of HF was successfully validated in the second independent population. Besides, the in vitro experiments revealed that G allele carriers were defective in β2AR-coupled inhibitory adenylate cyclase g (G_i_) protein signaling.

## Introduction

There is currently a global epidemic of heart failure (HF), with over 915,000 newly diagnosed patients with HF each year in the United States^1^. This complex syndrome is characterized by an interplay among genetic, neurohormonal, inflammatory, and metabolic factors^2,3^. HF therapy has been improved greatly over the past two decades, particularly with the use of angiotensin-converting enzyme inhibitors and β1-adrenergic receptor (β1AR) antagonists, which inhibit pathological remodeling and apoptosis of cardiomyocytes^4,5^. The current American College of Cardiology (ACC)/American Heart Association (AHA) guidelines recommend nondiscriminatory prescription of β-blockers to all stable HF with reduced ejection fraction (HFrEF), except for patients with comorbid contradictory conditions or who are unable to tolerate treatment with these drugs^6^. However, a substantial proportion of HF patients receive little or no benefit from the available therapeutics—often specifically owing to a lack of response to β-blockers^7–10^—and 5-year mortality is still >50%^1^. Changing official guidelines to include recommendations for patient stratification in the management of HF may open new avenues for the development of more effective and personalized therapies. For instance, new ways of identifying poor βAR-blocker responders before commencing treatment may revolutionize clinical practice and improve health management of HF patients.

During the progression of HF, the sympathetic nervous system becomes hyperactive. The resultant increase in β-AR stimulation to cardiomyocytes initially produces a positive inotropic effect, primarily via the activation of the β1AR-stimulating adenylate cyclase g (Gs) protein –adenylyl cyclase–cyclic adenosine monophosphate (cAMP)–protein kinase A signaling cascade^11^. However, persistent β1AR stimulation triggers apoptosis of cardiomyocytes and leads to hypertrophy, fibrosis and maladaptive remodeling of the diseased hearts, via mechanisms that depend on calcium/calmodulin-dependent kinase type II (CaMKII), but not on protein kinase A^12,13^.

β1AR is the predominant βAR subtype in the heart and the major mediator of the positive inotropic action of catecholamines under physiological conditions. During HF, however, β1AR is markedly desensitized and its expression is selectively downregulated. Consequently, the β1AR:β2AR ratio decreases from 4:1 in the normal heart to 3:2 in the failing heart, making the role of the β2AR subtype more important in the failing heart^14^. In contrast to β1AR that couples only to G_s_ proteins, β2AR couples to both G_s_ and inhibitory adenylate cyclase g (G_i_) proteins^15^. β2AR, therefore, has a distinct role in the heart. This becomes especially relevant under the pathological conditions of HF in which the expression of G_i_ proteins is elevated in the absence of alteration in the expression of G_s_ proteins^16,17^. Specifically, β2AR-Gs mediated contractile support in failing heart while the activation of β2AR-coupled G_i_ signaling protects the heart against various insults-induced loss of cardiomyocytes via the G_i_–Gβγ–phosphatidylinositol 3-kinase (PI3K)–AKT cell survival pathway^18,19^.

It is unclear how genetic factors contribute to β2AR signaling pathway and/or the differences in response to β-blockers in HF patients. To address this important question, we genotyped key genes involved in the βAR signaling pathway—*ADRB1*, *ADRB2*, *GRK2*, and *GRK5*—in blood samples from patients with HF and healthy control individuals. We then assessed the relationship between the identified gene variants and the prognosis of HF in a large cohort of patients with or without β-blocker treatment. In vitro experiments were designed to assess the signaling properties of the key identified variants.

## Results

### Resequencing data

By resequencing *ADRB1*, *ADRB2*, *GRK2*, and *GRK5* genes in blood samples from 100 pairs of patients with HF and healthy individuals, five missense variants—*ADRB1* Arg389Gly C > G, *ADRB2* Arg16Gly A > G, *ADRB2* Gln27Glu C > G, *GRK5* Gln41Leu A > T, and *GRK5* Arg304His G > A—and six synonymous variants were detected in the candidate genes (Supplementary Table S1); none of these single nucleotide polymorphism (SNPs) were located within *GRK2*. For all 11 SNPs identified, the minor allele frequency did not differ significantly between the HF and control groups, suggesting that the polymorphisms were not associated with the risk of HF.

### Patients and follow-up

**Fig. 1:**
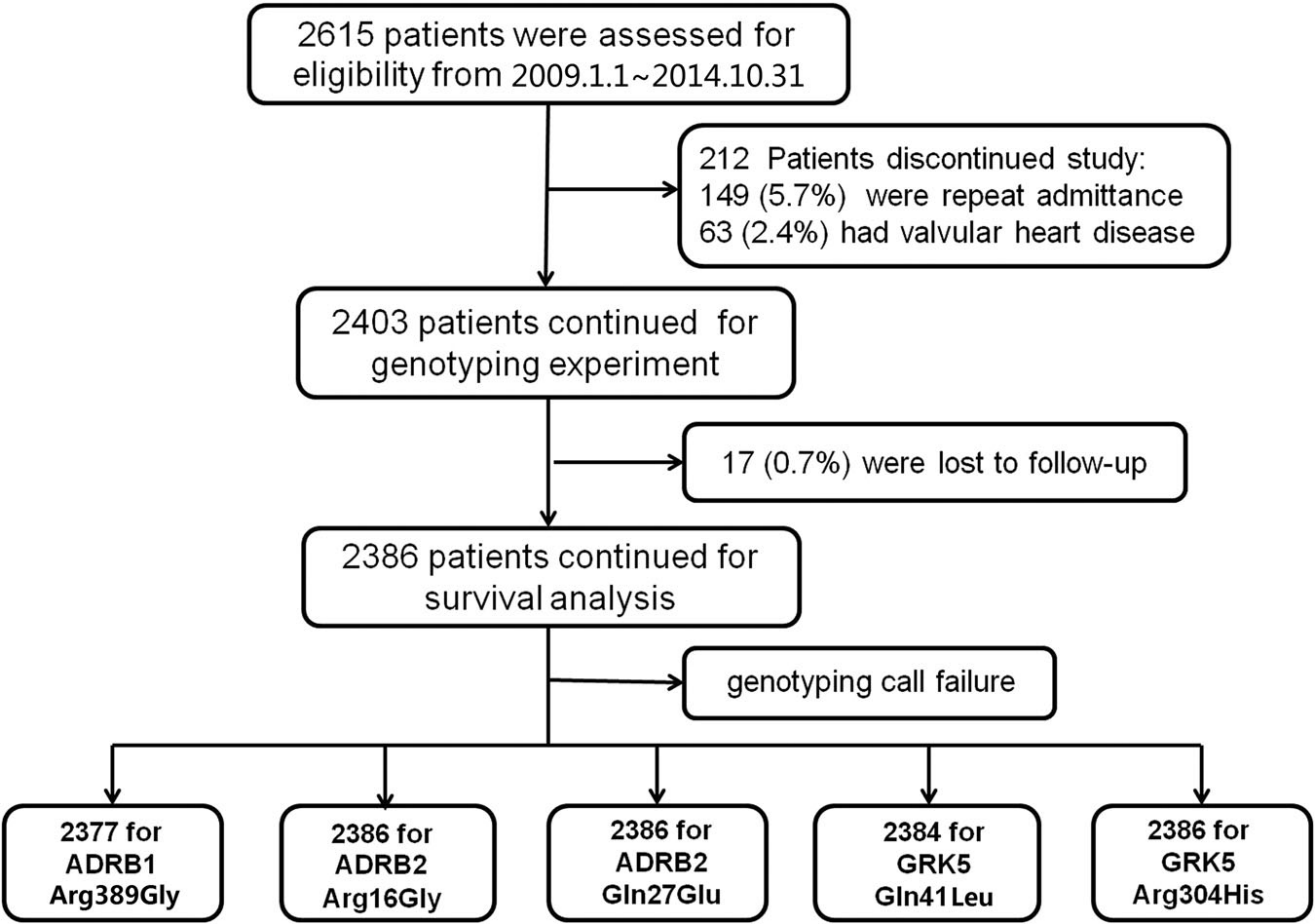
Enrollment and follow-up of patients with HF in the first stage. At study termination on July 2015 in the first discovery stage, 2615 patients with HF had been screened for eligibility to be included in the study in three centers in China and 2403 patients were finally enrolled and genotyped. Of these patients, 149 (5.7%) were excluded for repeat admittance, and 63 (2.4%) were excluded because valvular heart disease was considered the main reason for HF. Of the study participants, 17 were lost from follow-up because incorrect contact information had been provided on enrollment. Of the 2386 patients included in the outcome analysis: all were included in the analysis of the prognostic utility of the *ADRB2* Arg16Gly, *ADRB2* Gln27Glu, and *GRK5* Arg304His variants; 2377 patients were included in the analysis of the prognostic utility of the *ADRB1* Arg389Gly variants; and 2384 patients were included in the analysis of the prognostic utility of the *GRK5* Gln41Leu variants

A total of 2615 HF patients were recruited between 1 January 2009 and 31 October 2014 in the first cohort. Of the patients recruited, 212 were excluded from enrollment owing to repeat admittance (for 149 patients; 5.7% of all recruited) or valvular heart disease (in 63 patients; 2.4% of all recruited). Accordingly, 2403 patients were genotyped (Fig. 1). Among the overall study population (mean age 59.3 ± 14.5 years, 66.6% male), 56.6% had severe cardiac symptoms (New York Heart Association, (NYHA) class III or IV); 87.5% had a history of hypertension; 24.8% had a history of hyperlipidemia; and 31.8% had diabetes (Table 1). When study participants were stratified by genotype of the *ADRB2* Arg16Gly A > G locus (AA, AG, and GG), baseline characteristics did not differ significantly among the three groups (Table 1), except for hypertension, which was slightly less prevalent among patients with the AA genotype (84.4% for AA genotype group, 89.6% for the AG genotype group, and 89.1% for the GG genotype group; *P* < 0.001).

**Table 1:** Clinical characteristics of the patients by *ADRB2* Arg16Gly A > G genotype

Characteristics	All patients	Patients with AA genotype	Patients with AG genotype	Patients with GG genotype	*P* value^a^
	(*N* = 2403)	(*N* = 923)	(*N* = 1122)	(*N* = 358)	
Age (years)	59.3 ± 14.5	58.7 ± 14.5	59.0 ± 14.6	59.8 ± 14.5	0.208
Male sex^b^	1601 (66.6)	616 (66.7)	752 (67.0)	233 (65.1)	0.791
Ischemic etiology^b^	1137 (47.3)	450 (48.8)	525 (46.8)	162 (45.3)	0.343
NYHA functional class^b^					
II	1042 (43.4)	417 (45.2)	479 (42.7)	146 (40.8)	
III	812 (33.8)	316 (34.2)	379 (33.8)	117 (32.7)	0.201
IV	549 (22.8)	190 (20.6)	264 (23.5)	95 (26.5)	
Personal history^b^					
Smoking	937 (39.0)	365 (39.5)	436 (38.9)	136 (38.0)	0.87
Hypertension	2103 (87.5)	779 (84.4)	1005 (89.6)	309 (89.1)	<0.001
Hyperlipidemia	597 (24.8)	241 (26.1)	265 (23.6)	91 (25.4)	0.415
Diabetes	765 (31.8)	290 (31.4)	353 (31.5)	122 (34.1)	0.614
Stroke	246 (10.2)	102 (11.1)	113 (10.1)	31 (8.7)	0.434
Clinical testing					
Systolic pressure (mm Hg)	131.2 ± 25.2	131.8 ± 25.2	129.0 ± 24.2	131.3 ± 25.5	0.243
Diastolic pressure (mm Hg)	80.3 ± 16.2	80.7 ± 16.8	78.6 ± 14.4	80.5 ± 16.2	0.1
Heart rate (beats/min)	83.2 ± 20.2	82.7 ± 20.0	83.5 ± 20.0	83.6 ± 20.4	0.573
Serum creatinine (µmol/l)^c^	84 (67–106)	82 (66–105)	84 (69–107)	84 (66–107)	0.232
NT-proBNP (pg/ml)^c^	1857(424–5652)	1859 (514–5013)	1774 (405–5706)	2131 (351–6962)	0.435
LVEDD (mm)^c^	55 (48–64)	54 (47–63)	55 (48–64)	56 (47–64)	0.152
LAD (mm)^c^	40 (35–45)	39 (34–45)	40 (35–45)	40 (36–46)	0.2
Ejection fraction (%)^c^	47 (33–61)	48 (33–62)	47 (32–60)	44 (32–61)	0.334
Medication (%)					
Digoxin	563 (23.6)	214 (23.3)	274 (24.6)	75 (21.1)	0.382
Diuretics	1104 (46.3)	402 (43.7)	527 (47.4)	175 (49.3)	0.121
ACEI	1246 (52.2)	466 (50.7)	584 (52.5)	196 (55.2)	0.340
ARB	279 (11.7)	102 (11.1)	142 (12.8)	35 (9.9)	0.257
Beta-blocker	1365 (57.2)	534 (58.1)	636 (57.2)	195 (54.9)	0.590
Spironolactone	938 (39.3)	354 (38.5)	437 (39.3)	147 (41.4)	0.639

*ACEI* angiotensin-converting enzyme inhibitor, *ARB* angiotensin receptor blockers, *LAD* left atrial dimension, *LVEDD* left ventricular end-diastolic dimension, *NT-proBNP* the N-terminal pro-hormone of brain natriuretic peptide, *NYHA* New York Heart Association

^a^For similarity among the different genotypes

^b^Listed as number (%)

^c^Interquartile range included in parentheses

At the last assessment, the study follow-up compliance rate was 99.2% (2386/2403), and the mean follow-up period was 20.3 months (maximum: 60 months), with no significant difference among genotype groups (21.2, 19.6 and, 20.1 months for the AA, AG, and GG groups, respectively, *P* = 0.362). During follow-up, 57.2% of patients included in the outcome analysis were on βAR-blocker therapy (metoprolol or bisoprolol) for ≥6 months.

The basic characteristics of the patients in the replicated cohort were summarized in Supplementary Table S2 and the mean follow-up time was 15 months in the second cohort.

### Study outcomes for entire cohort in the first cohort

At the July 2015 cutoff date for the first cohort, cardiovascular death or heart transplantation (the primary end point) had occurred in 419 patients (17.6%). During follow-up, 448 (18.8%) patients died and 391 (16.4%) of them died from cardiovascular causes. Rehospitalization occurred in 929 (38.9%) patients, with 708 (29.7%) being rehospitalized for cardiovascular reasons. The recurrence of HF was observed in 628 (26.3%) patients, and 40 (1.7%) patients experienced new onset of stroke. The impact of conventional clinical risk factors on HF prognosis was assessed for the study cohort (Supplementary Table S3). Increasing age (hazard ratio (HR), 1.02; 95% confidence interval (CI), 1.01−1.03; *P* < 0.001), a history of diabetes (HR, 1.53; 95% CI, 1.26−1.86; *P* < 0.001), the etiology of the HF (nonischemic versus ischemic; HR, 1.28; 95% CI, 1.05−1.56; *P* = 0.013), NYHA functional class (class III + IV versus class II; HR, 3.04; 95% CI, 2.39−3.87; *P* < 0.001), and the level of serum NT-proBNP (HR, 1.55; 95% CI, 1.45−1.67; *P* < 0.001) were significantly and positively associated with the risk of the primary end point in our study.

**Fig. 2:**
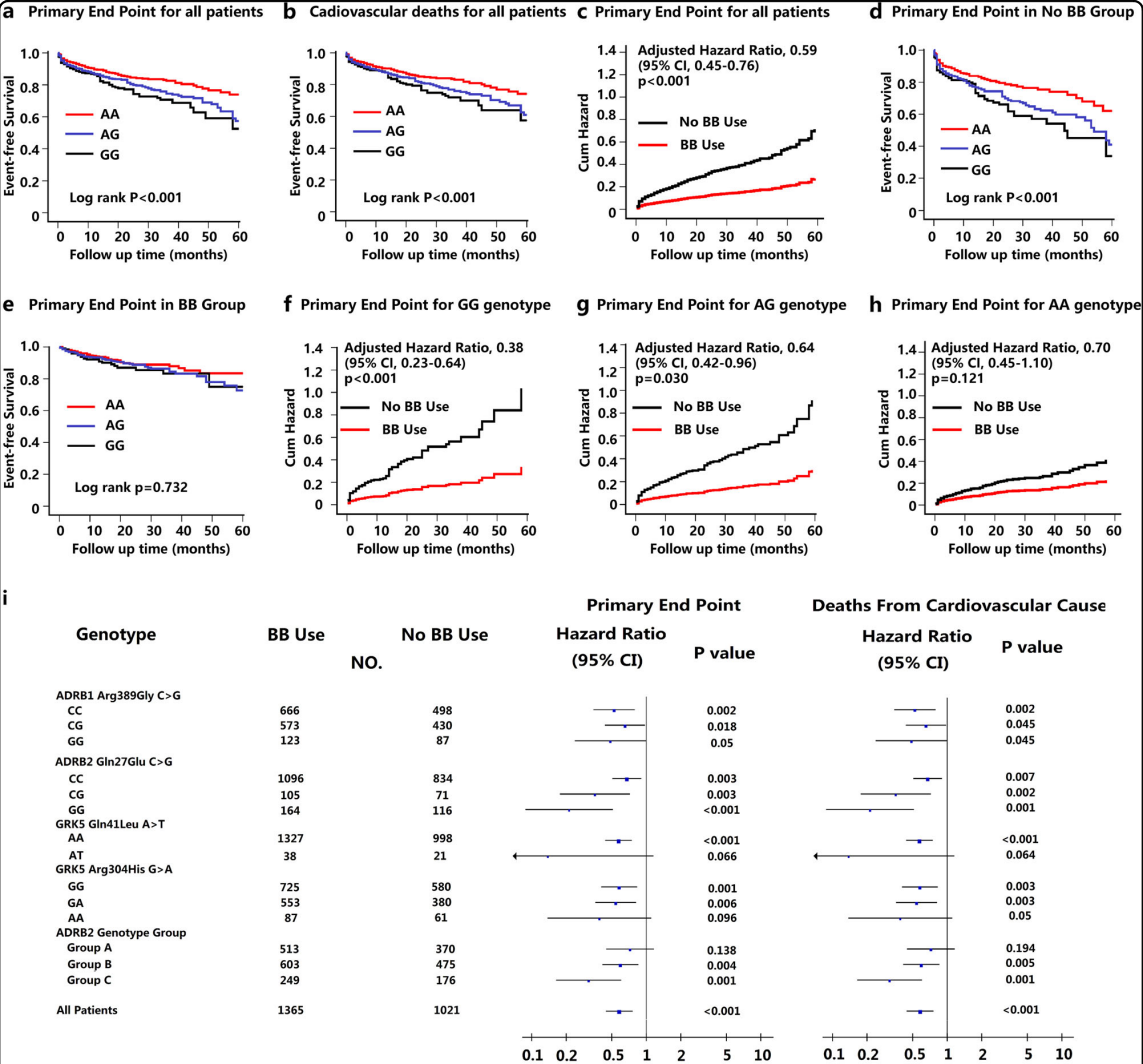
Clinical outcomes of heart failure patients and responses to β-blockers. **a** Kaplan−Meier curves of the primary composite end point showing that the clinical outcomes significantly varied among the groups of patients with the different genotypes at *ADRB2* amino acid site 16 (*P* < 0.001 by stratified log-rank test). G allele carriers had worse outcomes compared with those who were homozygous for AA (HR, 1.49; 95% CI, 1.21−1.83; *P* < 0.001 for AG/GG versus AA). **b** Kaplan−Meier curves of cardiac death demonstrating a similar association with the genotype of this SNP to the primary end point. The G allele was associated with increased risk of this individual end point (HR, 1.44; 95% CI, 1.16−1.79; *P* = 0.001 for AG/GG versus AA). **c** Among the entire cohort, β-blocker treatment was significantly associated with a reduced risk of the composite end point of cardiovascular death or heart transplantation (adjusted HR, 0.59; 95% CI, 0.45−0.76; *P* < 0.001). **d** For the patients who were not using BB therapy, Kaplan−Meier curves for the primary end point showed that the *ADRB2* Arg16Gly genotype was significantly associated with reduced transplantation-free survival (*P* < 0.001 by log-rank test). **e** For the patients who received BB therapy for ≥6 months during the study, the genotype-based heterogeneity was not significantly different (log-rank *P* = 0.732). The probability of the composite primary end point (cardiovascular death or heart transplantation) was significantly decreased with β-blocker use among patients with **f** the GG genotype (adjusted HR, 0.38; 95% CI, 0.23−0.64; *P* < 0.001) and **g** the AG genotype (adjusted HR, 0.64; 95% CI, 0.42−0.96; *P* = 0.03), but not with **h** the AA genotype (adjusted HR, 0.70; 95% CI, 0.45−1.10, *P* = 0.121). The extent of benefit of β-blocker treatment seemed to be G allele-dose-dependent. **i** Adjusted HRs are shown for the composite primary end point and for the individual end point of death from a cardiovascular cause for patients in the five specified missense variant subgroups. To assess the impact of both of the *ADRB2* missense polymorphisms together, we also examined outcomes for patients stratified according to three genotype combinations: homozygous for both Arg16 and Gln27 (that is, patients with only the major alleles; designated as group A), homozygous for both Gly16 and Glu27 (that is, patients with only the minor alleles; designated as group C), and other genotypes (designated as group B). The blue squares and black lines represent the HRs and 95% CIs. The size of the blue square corresponds to the number of patients in the subgroup. The *P* values were calculated by Cox proportional hazard models, with a two-sided *P* value of 0.05 indicating statistical significance after adjustment for the clinical covariants, with an unbalanced distribution between groups. BB β-blocker, CI confidence interval, HR hazard ratio

Of the five missense variants we identified in the exons of the *ADRB1*, *ADRB2*, and *GRK5* genes, only *ADRB2* Arg16Gly A > G (G being the minor allele, with a frequency of 38.2% in our HF cohort) was significantly associated with heterogeneity in the primary end point and an individual end point of cardiovascular deaths (both *P* < 0.001 by Log rank test) (Fig. 2a, b). The primary end point—cardiovascular death or heart transplantation—occurred in 129 (14.0%) patients with the AA genotype, compared with 209 (18.8%) patients with the AG genotype (HR, 1.42; 95% CI, 1.14−1.77; *P* = 0.002 for AG versus AA) and 81 (22.8%) patients with the GG genotype (HR, 1.22; 95% CI, 0.94−1.57; *P* = 0.136 for GG versus AG and HR, 1.71; 95% CI, 1.30–2.26; *P* < 0.001 for GG versus AA, respectively). By combining the patients with AG and GG genotypes into a single group, we found that the patients carrying G allele had a 50% higher risk of cardiovascular death or heart transplantation (HR, 1.49; 95% CI, 1.21−1.83; *P* < 0.001 for AG/GG versus AA; Table 2). However, when the analyses were conducted on patient subgroups with reduced or preserved left ventricular ejection fraction, the genotype-dependent variability in prognosis was only detected in the subgroup of HFpEF (heart failure with preserved ejection fraction) but not in the HFrEF subgroup (Supplementary Fig. S1).

**Table 2:** Primary, secondary, and other related outcomes, according to *ADRB2* Arg16Gly (A > G)

Outcome	AA genotype	AG genotype	GG genotype	HR^a^ (95% CI) or OR^b^	*P* value
	(*N* = 919)	(*N* = 1112)	(*N* = 355)	AG/GG vs. AA	
Primary composite outcome—no. (%)					
Death from cardiovascular causes or heart transplantation	129 (14.0)	209 (18.8)	81 (22.8)	1.49 (1.21–1.83)	<0.001
Death from cardiovascular causes	124 (13.5)	193 (17.4)	74 (20.8)	1.44 (1.16–1.79)	0.001
Secondary outcomes—no. (%)					
Death from any cause	138 (15.0)	226 (20.3)	84 (23.7)	1.50 (1.22–1.83)	<0.001
First hospitalization for cardiovascular causes	243 (26.4)	355 (31.9)	110 (31.0)	1.26 (1.08–1.48)	0.003
First hospitalization for any cause	317 (34.1)	482 (43.3)	130 (36.6)	1.30 (1.13–1.49)	<0.001
Recurrence of heart failure	228 (24.8)	304 (27.3)	99 (27.9)	1.15 (0.98–1.36)	0.086
Other related outcomes—no. (%)					
New onset of stroke	12 (1.3)	23 (2.1)	5 (1.4)	1.41 (0.72–2.78)	0.320
Improvement in NYHA class	505 (55.0)	557 (50.1)	170 (47.9)	1.22 (1.03–1.45)	0.021

*NYHA* New York Heart Association

^a^HR, hazard ratios and *P* value were calculated with the use of stratified Cox proportional hazard models adjusting with gender, age, and history of hypertension

^b^Improvement in heart function was analyzed as a binary outcome with the use of logistic regression model to calculate the odds ratio and *P* value

During follow-up, 15.0% (138/919) of patients in the AA group, 20.3% (226/1112) of patients in the AG group, and 23.7% (84/355) of patients in the GG group died (HR, 1.50; 95% CI, 1.22–1.83; *P* < 0.001 for AG/GG versus AA; Table 2). Most of these deaths (124 in the AA group, 193 in the AG group, and 74 in the GG group) had cardiovascular causes and the G allele was associated with increased risk of this individual end point (HR, 1.44; 95% CI, 1.16−1.79; *P* = 0.001 for AG/GG versus AA; Table 2). The heterogeneity in all-cause mortality, rehospitalization rate and improvement in NYHA class were similarly associated with the genotypes of this SNP. The recurrence of HF did not differ among the three genotype groups (Table 2).

Among the other four missense variants in the *ADRB1*, *ADRB2*, and *GRK5* genes, only the AT genotype of *GRK5* Gln41Leu (A > T, with a minor allele frequency of 1.2% in our study) was associated with an altered risk of any of the end points assessed. Patients with the AT genotype of *GRK5* Gln41Leu had a lower rate of cardiovascular rehospitalization (29.9% in the AA group versus 18.6% in the AT group, *P* = 0.029; no one in our cohort had the TT genotype) rather than the primary end point and all-cause death (Supplementary Table S4).

### Effects of β-blocker treatment on the prognosis of heart failure

Consistent with findings from previous clinical trials^20–22^, βAR-blocker therapy was associated with a reduced risk of the composite primary end point (adjusted HR, 0.59; 95% CI, 0.45−0.76; *P* < 0.001); cardiovascular death or heart transplantation occurred in 10.9% (149/1365) of patients taking βAR-blocker therapy and 26.4% (270/1021) of patients who were naïve to β-blockers (Fig. 2c). The risk of the individual end point of death from a cardiovascular cause was similarly reduced among patients taking βAR blockers (adjusted HR, 0.58; 95% CI, 0.44−0.76; *P* < 0.001) (Supplementary Fig. S2a); however, we detected no association between βAR-blocker therapy and rate of rehospitalization owing to a cardiovascular cause in our study (*P* = 0.788) (Supplementary Fig. S2b).

In the absence of a βAR-blocker intervention, transplantation-free survival (that is, the primary end point of the study) varied significantly among the groups stratified by the *ADRB2* Arg16Gly genotypes. Patients with the G allele were more likely to experience the primary end point (35.6% for patients with GG genotype, 29.8% for patients with AG genotype, and 19.4% for the patients with AA genotype; Log rank *P* < 0.001) (Fig. 2d). The outcome heterogeneity was not observed among the patients who were receiving βAR-blocker therapy (log-rank *P* = 0.732) (Fig. 2e), indicating that genetic variation may influence the efficacy of β-blocker treatment.

When multivariate analysis was performed using Cox proportional hazards models, the benefits of β-blocker treatment were confirmed to vary among the different genotypes of *ADRB2* Arg16Gly site. A pretest on the distribution of clinical characteristics stratified by β-blocker usage and various genotypes was conducted (Supplementary Tables S5–10). Notably, for ADRB2 Arg16Gly A > G site, βAR-blocker therapy improved the transplantation-free survival in patients with GG and AG genotypes in a G allele-dose-dependent manner (adjusted HR, 0.38; 95% CI, 0.23−0.64; *P* < 0.001 in the GG genotype group; and adjusted HR, 0.64; 95% CI, 0.42−0.96; *P* = 0.030 in the AG genotype group) (Fig. 2f, g and Supplementary Fig. S3). However, after adjustments, no significant effect of βAR-blocker therapy was observed in the AA genotype subgroup (adjusted HR, 0.70; 95% CI, 0.45−1.10; *P* = 0.121; Fig. 2h, Supplementary Fig. S3). After combining AG and GG into one group, the risk of cardiovascular death or heart transplantation was decreased by approximately 50% when β-blockers were used (adjusted HR, 0.51; 95% CI, 0.38−0.70; *P* < 0.001). Beneficial therapeutic effects of β-blocker and the *ADRB2* Arg16Gly site-associated heterogeneities in β-blocker responses were consistent in both HFrEF and HFpEF subgroups (Supplementary Fig. S4).

Apparent heterogeneities in the response to β-blockers were also detected for some of the other SNPs (Fig. 2i). In patients with AT genotype of the *GRK5* Gln41Leu A > T site (59 patients, 2.5% of all patients) or the AA genotype of *GRK5* Arg304His G > A site (148 patients, 6.2% of all patients), β-blocker treatments were not significantly associated with a reduction in the risk of cardiovascular death or heart transplantation (*P* = 0.066 and *P* = 0.096, respectively), unlike in patients with the other variants at these loci.

Although a beneficial response to β-blockers was observed in all three genotypes at the *ARDB2* Gln27Glu C > G site, these beneficial effects were increased in a G allele-dose-dependent manner (adjusted HR, 0.66; 95% CI, 0.50−0.87; *P* = 0.003 for the CC group; adjusted HR, 0.36; 95% CI, 0.18−0.71; *P* = 0.003 for the CG group; and adjusted HR, 0.25; 95% CI, 0.11−0.57; *P* < 0.001 for the GG group) (Fig. 2i). To assess the impact of both of the *ADRB2* missense polymorphisms together, we examined outcomes in study participants grouped on the basis of their status with regards to the two polymorphisms. The benefits associated with βAR-blocker therapy when patients were stratified according to three genotype combinations—homozygous for both Arg16 and Gln27 (that is, patients with only the major alleles; designated as group A), homozygous for both Gly16 and Glu27 (that is, patients with only the minor alleles; designated as group C), and other genotypes (designated as group B)—were analyzed. βAR-blocker treatment was associated with a significantly decreased risk of the primary end point in the patients with at least one minor allele (adjusted HR, 0.60; 95% CI, 0.42−0.85; *P* = 0.004 for genotype group B; and adjusted HR, 0.33; 95% CI, 0.17−0.61; *P* = 0.001 for genotype group C), but not in patients with no minor allele (adjusted HR 0.70; 95% CI, 0.44−1.12; *P* = 0.138 for genotype group A; Fig. 2i).

### ADRB2 Arg16Gly-dependent clinical outcomes in the replicated cohort

To further substantiate our findings from the first cohort, we subsequently carried out an independent replication study by genotyping another cohort of 919 chronic HF patients to validate the observed associations. We successfully replicated the ADRB2 Arg16Gly site-dependent heterogeneity in prognosis of HF in this independent cohort, and the result showed that in the natural state without treatment of β-blockers, patients carrying G allele (AG/GG genotype) had worse prognosis compared with patients who are AA homozygous (adjusted HR, 2.09; 95% CI, 1.06−4.12; *P* = 0.034 for AG/GG versus AA; Supplementary Table S11). We also reinvestigated the response to βAR-blocker therapy in different genotype group according to ADRB2 Arg16Gly site. The results confirmed that, after adjusting for age, sex, NYHA functional class, and other drug treatments, G allele carriers are good responders to β-blockers (adjusted HR, 0.86; 95% CI, 0.31−2.42; *P* = 0.774 for AA genotype group; adjusted HR, 0.49; 95% CI, 26−0.92; *P* = 0.026 for AG/GG genotype group, Supplementary Table S11).

### Effects of *ADRB2* Arg16Gly variation on β2AR function in vitro

**Fig. 3:**
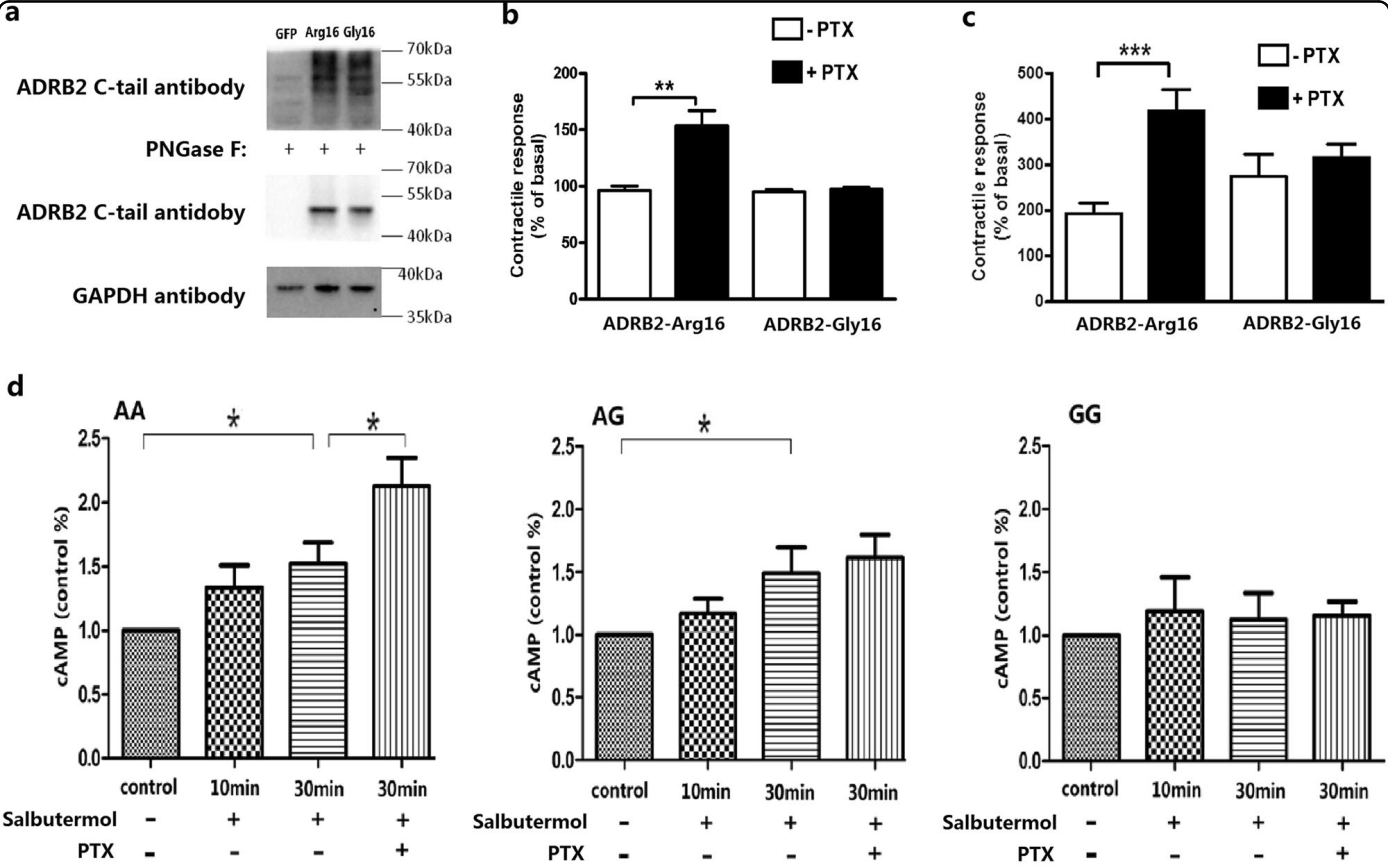
Functional analysis of the *ADRB2* Arg16Gly variants in cell culture systems. **a** The western blots of β2AR and GAPDH for lysates of adenovirus-infected rat cardiomyocytes showing that the transfection method (multiplicity of infection of 100 for 24 h) produced equal expression of *ADRB2*-Arg16 and *ADRB2*-Gly16, which is true irrespective of deglycosylation treatment of the cell lysates with PNGase F. **b** PTX treatment (0.75 μg/ml overnight) enhanced the zinterol (1×10^−5^ M)-induced contractile response in adult rat cardiac myocytes expressing *ADRB2*-Arg16, but not in those expressing *ADRB2*-Gly16 (multiplicity of infection of 100 for 24 h; *N* = 8 for each group). **c** *ADRB2* knockout mouse cardiomyocytes were infected with Adeno-*ADRB2*-Arg16 or Adeno-*ADRB2*-Gly16 viruses as described in Materials and Methods and then subjected to stimulation with zinterol (3×10^−7^ M). The zinterol-induced contractile responses were 200−300% in the absence of PTX. PTX enhanced these responses in cells expressing *ADRB2*-Arg16, but not in cells expressing *ADRB2*-Gly16. (*N* = 10–16 cells from four mouse hearts for each data point). **d** Peripheral lymphocytes were isolated from HF patients with different genotypes at the *ADRB2* Arg16Gly locus. The inhibition of G_i_ by PTX enhanced β2AR-mediated cAMP accumulation in cells with the AA genotype (*N* = 10; *P* < 0.05), whereas cAMP accumulation in cells with either the AG (*N* = 10) or GG (*N* = 6) genotype was insensitive to PTX treatment. Therefore, peripheral lymphocytes harboring the G allele of the *ADRB2* Arg16Gly A > G polymorphism showed deficient coupling of G_i_ to the β2AR. All the data were shown as the mean ± SEM. β2AR β2 adrenergic receptor, HF heart failure, PTX pertussis toxin. *p<0.05, **p<0.01, ***p<0.001

To explore the possible mechanism underlying the relationship between the *ADRB2* Arg16Gly polymorphism and long-term prognosis and response to βAR-blocker therapy, we examined the downstream signaling events of different β2AR genotypes in primary cultured rodent myocardial cells and human peripheral lymphocytes. Transduction efficiency of the control Adeno-GFP virus was over 90% in our preliminary test on adult rat cardiomyocytes (Supplementary Fig. S5), similar to our previous reports^13,23^. When rat cardiomyocytes were infected with one of the Adeno-*ADRB2*-Arg16, Adeno-*ADRB2*-Gly16 or Adeno-GFP viruses, we found successful expression of the two β2AR variants at comparable levels (Fig. 3a and Supplementary Fig. S6). Disruption of G_i_ signaling with pertussis toxin (PTX) significantly augmented the contractile response to zinterol, a β2AR agonist, in adult rat cardiomyocytes expressing human *ADRB2*-Arg16, but not in cells expressing *ADRB2*-Gly16 (Fig. 3b). Furthermore, in *ADRB2* knockout mouse cardiomyocytes infected with Adeno-*ADRB2*-Arg16 or Adeno-*ADRB2*-Gly16 and without PTX pretreatment, zinterol increased cell contractility to 200–300% of the basal levels (Fig. 3c, Supplementary Fig. S7). PTX treatment markedly enhanced zinterol-induced contractile response in cells expressing *ADRB2*-Arg16, but not in cells expressing *ADRB2*-Gly16 (Fig. 3c). Similar results were observed in lymphocytes isolated from HF patients with the various β2AR genotypes: the inhibition of G_i_ signal by PTX significantly enhanced the β2AR-mediated elevation of cAMP levels in lymphocytes with the AA genotype (*P* < 0.05), whereas cells with AG or GG genotype were insensitive to PTX treatment (Fig. 3d). Thus, the results indicate that the *ADRB2* Arg16Gly is a loss-of-function polymorphism with respect to G_i_ protein-coupling in all of the used experimental systems.

## Discussion

In the first cohort of this study, a multicenter observational study of 2403 patients with chronic HF reveals that only Arg16Gly A > G polymorphism in *ADRB2* gene seems to influence the prognostic outcome of HF. The G allele is independently associated with a nearly 50% increase in the risk of cardiovascular death or transplantation after HF. Furthermore, we have shown that chronic HF patients carrying the *ADRB2*-Gly16 allele (AG or GG genotype) have a markedly increased beneficial response to βAR-blocker therapy in an allele-dose-dependent manner. In all, 36 and 62% reductions of risk in the composite end point of cardiovascular death or heart transplantation were observed for the AG (*P* = 0.03) and the GG genotype (*P* < 0.001), respectively (Fig. 2f, g). In contrast, after multiple adjustments, the patients with the AA genotype experienced no significant benefit from βAR-blocker therapy (Fig. 2h). These observations can be successfully replicated in a separate chronic HF patient cohort. The influence of the G allele on the efficacy of βAR-blocker therapy is independent of conventional treatments, including angiotensin-converting enzyme inhibitors, spironolactone, digoxin, and diuretics with multiple adjustments. These findings provide a molecular basis among others for the long-standing question in the cardiovascular field of why nearly half of the HF patients do not respond well to β-blocker therapy^24–27^.

Mechanistically, we have demonstrated that disruption of G_i_ signaling with PTX cannot alter β2AR-mediated contractile response in cardiomyocytes expressing *ADRB2*-Gly16, but markedly enhances the positive inotropic effect of β2AR stimulation in cardiomyocytes expressing *ADRB2*-Arg16 in both adult rat model and β2AR-deficient mouse model (Fig. 3b, c). This conclusion is corroborated by the fact that PTX potentiates β2AR-stimulated cAMP production in human lymphocytes carrying two *ADRB2*-Arg16 alleles but not in cells expressing *ADRB2*-Gly16 (Fig. 3d). Taken together, we have demonstrated that β2ARs encoded by *ADRB2*-Gly16 cannot couple to G_i_, while wild-type β2ARs (encoded by *ADRB2*-Arg16) activate both G_s_ and G_i_ signaling pathways.

In HF, the hyperactivity of the sympathetic nervous system with an excessive βAR stimulation—the salient feature of this syndrome—is widely considered to be a compensatory mechanism for impaired heart function^16,28^. In the acute phase, activated βAR signaling is beneficial to meet the demands of the body; however, chronic βAR stimulation, especially β1AR persistent activation, initiates a cascade of cardiac hypertrophy and apoptosis via evoking CAMKII signaling pathway^11^. β1AR and β2AR are coexpressed in the heart, but exhibit distinct functions under certain pathological circumstances, such as chronic HF. Unlike the β1AR, which only couples to G_s_, β2AR also couples to PTX-sensitive G_i_ proteins^15^ and mediates cardioprotective effects through the G_i_–Gβγ–PI3K–AKT cell survival pathway^18,19^. Previous studies have shown that the deficiency of β2AR enhances isoproterenol- or doxorubicin-induced myocardial injuries and mortality in mice^29,30^, and the loss-of-function *ADRB2* Thr164Ile mutation is associated with increased mortality in patients with HF^31^. These outcomes are likely the consequence of the loss of the β2AR-G_i_–Gβγ–PI3K–AKT cell survival pathway^18,19^. Meanwhile, β2AR-Gi signaling has been reported to mediate the crosstalk of β adrenergic receptor subtypes. Specifically, β2AR-G_i_ signaling abrogates β1AR-induced loss of cardiomyocytes and negates both β1AR-mediated and β2AR-mediated positive inotropic effects by negating the activation of l-type calcium channel and CaMKII^32–35^. Thus, β2AR-evoked G_i_ signaling biologically mimics the β1AR blocking effect. In the present study, we have demonstrated that *ADRB2*-Gly16 is deficient in its coupling to G_i_ proteins (loss of function), which resulted in a loss of endogenous protection. In addition, the deficiency of the β2AR-G_i_-signaling may exaggerate the pathogenic β1AR signaling. Thus, in the absence of βAR-blocker treatment, the subgroups of patients with AG or GG genotype have a markedly worse prognosis compared with patients with the AA genotype. Meanwhile, the reduced inhibition of the β1AR-G_s_ signaling by the β2AR-G_i_ in *ADRB2*-Gly16-expressing myocardial cells may explain why the G allele carriers are hypersensitive to βAR-blocker therapy. Moreover, we and others have demonstrated that selective activation of β2AR-coupled G_s_ with fenoterol is also beneficial in multiple HF models^36,37^, and that β2AR-mediated G_s_ signaling provides contractile support without evoking the CaMKII-dependent cell death signaling cascade^38^. Thus, finely tuned β2AR-coupled G_s_ and G_i_ signaling has important pathological and therapeutic implications in HF. Especially for patients who are insensitive to β1AR blockade therapy, β_2_-AR activation treating might be a potential novel therapy in future^39^.

Notably, several previous studies have been conducted to investigate the association between *ADRB2* polymorphism and HF prognosis; however, the results are lack of enough power and not consistent^31,40,41^. The reason is mainly due to racial differences in the study populations. While exclusively Han Chinese are enrolled in the present study, the major composition of the study populations in these previous studies is Caucasian (>70%)^31,40,41^. For the *ADRB2* Arg16Gly A > G variant, the G allele is the minor allele in the Chinese Han population, with a frequency of 38.2% in our study, whereas it is the major allele in the European population (61.4%, 1000 Genomes Project Phase 3: http://asia.ensembl.org/Homo_sapiens/Variation/Population?db=core;r=5:148826377-148827377;v=rs1042713;vdb=variation;vf=747520)^42^. Moreover, AA homozygotes (38.4% in our study), which have poor response to β-blockers, exhibit only a frequency of 13.5% in the European population^42^. The nonresponsiveness to β-blocker therapy was not readily detected in previous studies, because those studies enrolled large proportions of Caucasians, which had a low frequency of AA homozygotes^31,40,41^. In addition, small sample sizes (500 for one study and 186 for another) and limited follow-up period (12 months) in some studies also partly explain why previous studies have failed to reveal this association^40,41^. Differences in inclusion criteria may also contribute to the observed inconsistency between this and previous studies. For instance, most previous studies have only included HF patients with reduced ejection fraction (<35–40%)^20–22^, whereas our study included both HFrEF and HFpEF subjects.

In addition to the *ADRB2* Arg16Gly variant, previous studies have shown the contributions of other naturally existing SNPs in related genes to the heterogeneity of HF patients to β-blocker therapy^9,24–27,43^. In particular, for the *ADRB1* Arg389Gly C > G variant, we have shown that homozygotes *ADRB1-*Arg389 in our HF cohort exhibit a better response to βAR-blocker treatment (HR, 0.52; *P* = 0.002) than Gly389 homozygotes (HR, 0.51; *P* = 0.05) (Fig. 2i), similar to the situation of Caucasians^26^. Furthermore, HF patients harboring one or two Glu27 alleles in the *ADRB2* Gln27Glu locus (the G allele) exhibit better responses to β-blockers than Gln27 (the C allele) homozygotes (HR, 0.25 for the GG genotype; HR, 0.36 for the CG genotype; and HR, 0.66 for the CC genotype) (Fig. 2i), consistent with previous reports^41,43^.

For the *GRK5* Gln41Leu A > T locus, the T allele frequency is much higher in African American populations (32.6%) than in Chinese (1.1–1.3%) or Caucasians (2.6%) populations^25^. African American HF patients with the T allele exhibit a greater survival rate in the absence of β-blockers than those carrying the A allele, but they are resistant to βAR-blocker therapy^25,27^. However, in our study, among 59 patients carrying the T allele, only three patients with and five patients without βAR-blocker treatment experienced the primary end point event during follow-up. Thus, the present study is underpowered for this particular analysis.

Although clinical guidelines recommend maximal and nondiscriminatory prescription of β-blockers to all patients with stable HF in early stages, except for patients with comorbid contradictory conditions or who are unable to tolerate treatment with these drugs^6,44^, objective records indicate that only 57.2% of our patients (64.3% of patients with NYHA class II HF, 56.3% of patients with NYHA class III HF, and 45.1% of patients with NYHA class IV HF) received β-blocker treatment. These medication rates have enabled us to collect comprehensive information regarding how patient genotype affects the progression of HF without the medication. Many previous studies, which have not demonstrated an effect of the *ADRB2* SNPs on HF prognosis, have medication rates of 80−100% for β-blockers^45–48^. Here, we are able to show on the basis of our data and previous reports that the therapeutic responses to β-blockers actually vary among genotypic subgroups of HF patients^25–27^. Application of βAR-blocker therapy based on patient’s genotype should be strongly recommended.

In conclusion, in this large-scale, multicenter and observational study of HF, we have validated a strong association between the Arg16Gly A > G polymorphism in the *ADRB2* gene and clinical outcomes. The G allele carriers had a worse prognosis but responded well to β-blocker treatment. These findings provide both an explanation for the profound heterogeneity in the response to β-blocker therapy and the theoretical basis for individualizing β-blocker therapy in HF.

## Materials and methods

### Study oversight

The study was funded by the National Basic Research Program of China. The executive committee designed and oversaw the conduct of the study and data analysis. All protocols and methods were approved by the ethics committees of the local hospitals (Ethics Committee of Tongji Hospital) and conducted in accordance with the Declaration of Helsinki and the International Conference on Harmonization Guidelines for Good Clinical Practice. We obtained written informed consents from all participants. An independent data and safety monitoring committee periodically reviewed the study data.

### Study design

A multicenter observational study was designed to investigate the combined effects of genotype and β-blocker therapy on prognosis, without interfering with any routine treatment procedures for patients with HF. In the preparatory stage, sequencing data for 100 pairs of HF patients and healthy control individuals from the local Chinese Han population were obtained to determine the allele frequency and distribution of polymorphisms of genes involved in βAR signaling in this population. From January 2009, patients who were diagnosed as having HF were enrolled in this study at three centers in Southern (Tongji Hospital Affiliated to Tongji Medical Collage, Huazhong University of Science and Technology, Wuhan) and Northern (the Second Hospital of Hebei Medical University, Shijiazhuang; and Fuwai Hospital, National Center for Cardiovascular Disease, Beijing) China. A second separate cohort enrolled 919 patients with chronic HF and was used to validate the results observed in the first cohort. Patients were followed up periodically until the termination of the study. The primary end point was a composite of cardiovascular death or heart transplantation. The secondary end points included all-cause mortality, recurrence of HF, and first rehospitalization for cardiovascular causes. These rehospitalizations were defined as hospital admission for angina, arrhythmias, or HF-related symptoms, such as dyspnea or edema. Other related outcomes, such as hospitalization for any cause, new onset of stroke, and symptom relief as determined using the NYHA functional class, were also recorded during follow-up. The sample size and the number of required events were evaluated according to the preplanned interim analysis for the primary end point.

### Eligibility and study procedures

From January 2009 to October 2014, 2615 patients were screened for eligibility for admission to the study. The replicated cohort included 919 patients with chronic HF recruited in Tongji Hospital (Wuhan) from October 2014 to March 2016. Eligibility requirements included age ≥ 18 years, NYHA class II−IV HF with reduced or preserved left ventricular ejection fraction, and willingness to participate in long-term follow-up. The diagnosis of HF was confirmed by physical examination, laboratory tests, echocardiography, and according to the established protocols and criteria of the ACC and AHA^6^. At the time of study entry, detailed clinical data were obtained using a standardized questionnaire administered to the patients, with verification via medical records. The exclusion criteria were: severe valvular heart disease as the leading cause of HF; life-threatening complications, such as severe liver dysfunction, renal dysfunction, or a history of malignancy and life expectancy < 1 year; second or third degree atrioventricular block, unless the patient had received a pacemaker; acute myocardial infarction or unstable angina within 1 month before admission; or refusal to participate in the follow-up. Whether the patient was treated with β-blockers was left to the treating physician’s discretion. The investigators were instructed to make follow-up telephone calls or to perform face-to-face interviews at predesignated times after patient discharge, to collect relevant information on the clinical events under evaluation. Information regarding medication usage for therapies administered for >6 months was also collected. The duration of follow-up was defined as the interval from the date of enrollment to the date when the primary end point occurred or the last contact.

### Resequencing and genotyping

The DNA samples were prepared as previously reported^49^. The coding regions of the *ADRB1*, *ADRB2*, *GRK2*, and *GRK5* genes were screened via directional Sanger sequencing using the ABI 3130 BigDye Terminator v3.1 Cycle Sequencing Kit (Applied Biosystems, Foster City, CA, USA) on an ABI 3130xl sequencer. The primer sequences and amplicon sizes are given in Supplementary Table S12. The sequence analyses were performed using a 3130 Genetic Analyzer (Applied Biosystems, Foster City, CA, USA). The 7900 HT PCR system (Applied Biosystems, Foster City, CA, USA) and TaqMan allelic discrimination assay were used for genotyping. Forward and reverse TaqMan primers and FAM/VIC-labeled MGB probes, designed by Applied Biosystems or Shanghai GeneCore BioTechnologies Co. Ltd., were used for allelic discrimination (Supplementary Table S13), and the genotype assignments were performed using SDS Software v2.3.

### Adenoviruses

Culture and adenovirus-mediated gene transfer of adult rat or mouse cardiomyocytes were implemented by methods described previously^13,23^. The starting DNA sequence for the generation of vector constructs of adenoviruses was that codes for the wild-type (Arg16) human β2AR, with the following modifications: an N-terminal hemagglutinin tag followed by a FLAG sequence, and a C-terminal polyhistidine tag^50^. The shuttle vectors were generated by sub-cloning. Site-directed mutagenesis on the *ADRB2* gene sequence was performed with the QuickChange II site-directed mutagenesis kit (Agilent Technologies, Santa Clara, CA, USA) to introduce a single nucleotide change (A to G, Arg16Gly) to mimic the naturally occurring polymorphism. An adenovirus carrying the wild-type human *ADRB2*-Arg16 sequence, one carrying the minor allele sequence *ADRB2*-Gly16, and the control adenovirus for jellyfish green fluorescent protein (GFP) were amplified, purified, and titered using established protocols described previously^13,23^.

### Cardiomyocyte isolation, adenoviral infection, and contractility measurement

Cardiomyocytes were isolated from the hearts of Sprague-Dawley rats (male, 180–230 g) or *ADRB2* knockout mice (male, 2–4 months) using standard enzymatic techniques^13–15,23^. Cardiomyocytes were seeded on laminin-coated coverslips and infected with the appropriate adenovirus at a multiplicity of infection of 100. Subsequently, the myocytes were cultured for 24 h in medium supplemented with forskolin (10 μM) and 2,3-butanedione monoxime (10 mM) to reestablish G_i_ coupling of the β2AR expressed from the viral vector^23,51^.

For protein determination, adenovirus-infected rat cardiomyocytes (about 50,000 cells) were lysed in a lysis buffer (Solarbio Science & Technology, Beijing, China) containing a protease inhibitor mixture (Roche Diagnostics, Basel, Switzerland). Cell lysates were clarified by centrifugation. A portion of the samples for β2AR detection were further denatured and treated with peptide: *N*-glycosidase (PNGase F, New England Biolabs, Ipswich, MA, USA) for 2 h at 37 °C. The samples were denatured in Laemmli sample buffer and resolved by SDS-PAGE. The β2AR (C-tail antibody sc-569 from Santa Cruz Biotechnology, Santa Cruz, CA, USA, 1:1000) and GAPDH (antibody from EasyBio, China, 1:1000) were detected by immunoblotting. Densitometry was performed using NIH ImageJ. Ratios between the β2AR and the housekeeping protein GAPDH were calculated to normalize protein quantity.

Contractility measurements were performed as previously described^15^. Briefly, cardiomyocytes were perfused with a buffer containing 137 mM NaCl, 4.9 mM KCl, 1.2 mM MgCl_2_, 1 mM NaH_2_PO_4_, 1 mM CaCl_2_, 20 mM glucose, and 20 mM HEPES (pH 7.4), and electrically paced at ambient temperature on a microscopic stage. Cell length was monitored by an optical edge tracking method using an instrument setup manufactured by IonOptix (Milton, MA, USA). The measurements were made under steady-state conditions before and after treatment with a β2AR agonist, zinterol (from Tocris Bioscience, Bristol, UK; 10 μM for rat cardiomyocytes or 0.3 μM for *ADRB2* knockout mouse cardiomyocytes). In a subset of experiments, cells were pretreated with pertussis toxin (PTX, from List Biological Laboratories, Campbell, CA, USA; 0.75 μg/ml overnight) to block G_i_ signaling, as previously described^15,51^.

### Human peripheral lymphocyte separation and measurement of cAMP accumulation

Suspended lymphocytes from peripheral blood of HF patients with different genotypes of *ADRB2* Arg16Gly site (AA, AG, or GG) (Supplementary Table S14) were prepared using lymphocyte separation medium (LTS10770125, Tian Jin Hao Yang Biological Manufacture, China) according to the manufacturer’s instructions. The separated cells were seeded in six-well plates V (10^7^ cells/well) and cultured at 37 °C in RPMI-1640 medium (Flow, Rockville, MD, USA) containing 10% fetal bovine serum (Hyclone, Logan, UT, USA) and supplemented with 100 U/ml penicillin and 100 mg/ml streptomycin. G_i_ signaling was specifically disrupted by pretreatment with 1.5 µg/ml PTX for 3 h before stimulation with 10 μM salbutamol for 10 or 30 min. To inhibit the degradation of cAMP, 200 μM 3-isobutyl-1-methylxanthine was added to each well 5 min before stopping the reaction at indicated time points. The cell lysates were used in the cAMP assay according to the instructions for the enzyme-linked immunosorbent assay kit (R&D Systems, Minneapolis, MN, USA). The accumulation of cAMP in whole cell lysates was compared among different genotype groups.

### Statistical analysis

We conducted a preplanned interim analysis for survival free from heart transplantation, and the sample size and required number of primary outcome events were calculated on the basis of the genotype of the *ADRB2* Arg16Gly locus. Patients who were heterozygous or homozygous for Gly16 were combined into a single group, owing to the determined event rate. A 90% power to detect significant prognostic differences between G allele carriers and the AA homozygotes of *ADRB2* Arg16Gly polymorphism at an overall two-sided alpha level of 0.05 was desired. To achieve this outcome, with 344 effective events, we estimated that approximately 2700 patients would need to be followed up. However, the event rate was higher than expected. As of July 2015, 419 primary end points occurred in all patients enrolled in the first cohort, providing a power of 96% to detect a significant difference for this outcome. We therefore decided to terminate and selected July 2015 as the cutoff date for the final summary of the *ADRB2* Arg16Gly variant analysis in the first cohort.

The clinical baseline characteristics of the participants enrolled in two cohorts are presented as the means, standard deviations; medians, interquartile range for continuous variables and counts and percentages for categorical variables. Comparisons between two groups were performed by independent samples *t* test and chi-square test. Multiple comparisons were performed by one-way ANOVA using the post-hoc test. Time-to-event data were estimated using Kaplan−Meier analysis, and *P* values were evaluated based on log-rank tests stratified according to the SNP genotypes. HRs and 95% CIs for β-blocker treatment were estimated using Cox proportional hazards models. A two-sided *P* value of 0.05 indicated statistical significance after adjustment for the clinical covariants with an unbalanced distribution between the groups. Improvement in NYHA functional class from enrollment through the follow-up period was analyzed as a binary outcome (improved condition versus no change or deteriorated condition) using a logistic regression model.

Statistical analyses were performed using the SPSS statistical software package (Version 16.0, SPSS Inc., Chicago, IL, USA) or Prism 5.0 (GraphPad Software Inc, San Diego, CA, USA). All reported *P* values are two-sided, and those <0.05 were considered to be statistically significant.

## Electronic supplementary material

Supplementary Information

## Electronic supplementary material

**Supplementary Information** accompanies the paper at (10.1038/s41421-018-0058-6).
