# Supplementary material for: ADRB2 polymorphism Arg16Gly modifies the natural outcome of heart failure and dictates therapeutic response to β-blockers in patients with heart failure
Source: Cell Discov. 2018 Oct 23;4:57. doi: 10.1038/s41421-018-0058-6 (PMC6198009; doi:10.1038/s41421-018-0058-6)
Supplement: Supplementary file 1 — Supplementary Information [file 41421_2018_58_MOESM1_ESM.pdf]

Supplementary Information

Table S1. Frequency Distribution of β-AR Signaling Pathway Polymorphisms in Our Chinese Han Population Determined by Sequencing

Gene	AA change	Allele change	Type of variant	MAF in patients with HF (%)	MAF in healthy controls (%)
ADRB1	Arg389Gly	C>G	missense	18.8	25.0
ADRB2	Arg16Gly	A>G	missense	42.3	43.9
ADRB2	Gln27Glu	C>G	missense	12.5	9.8
ADRB2	Leu84Leu	G>A	synonymous	32.6	33.8
ADRB2	Arg175Arg	C>A	synonymous	30.7	33.3
ADRB2	Gly354Gly	G>C	synonymous	41.7	35.1
ADRB2	Leu413Leu	G>A/T	synonymous	41.1	31.9
GRK5	Gln41Leu	A>T	missense	2.1	2.6
GRK5	Ile62Ile	C>T	synonymous	4.6	5.1
GRK5	Asn280Asn	C>T	synonymous	6.3	3.4
GRK5	Arg304His	G>A	missense	18.8	25.0

Abbreviations: AA, amino acid; AR, adrenergic receptor; HF, heart failure; MAF, minor allele frequency.

Table S2. Clinical Characteristics of the patients in replicated stage

Characteristics	All patients (N=919)	Patients with AA genotype (N=324)	Patients with AG /GG genotype (N=595)	P Value*
Age (years)	61.0±12.7	60.8±12.4	61.0±12.8	0.377
Male sex[§]	582 (63.3)	212 (65.4)	370 (62.2)	0.329
NYHA functional class[§]				
II	474 (51.6)	166 (51.2)	308 (51.8)	
III	327 (35.6)	115 (35.5)	212 (35.6)	0.958
IV	118 (12.8)	43 (13.3)	75 (12.6)	
Personal history[§]				
Smoking	313 (34.1)	114 (35.2)	199 (33.4)	0.595
Hypertension	526 (57.2)	178 (54.9)	348 (58.5)	0.299
Hyperlipidemia	321 (34.9)	109 (33.6)	212 (35.6)	0.546
Diabetes	234 (25.5)	94 (29.0)	140 (23.5)	0.068
Stroke	74 (8.1)	26 (8.0)	48 (8.1)	0.982
Clinical testing				
Systolic pressure (mmHg)	133.3±24.4	134.6±24.7	132.5±24.3	0.366
Diastolic pressure (mmHg)	79.6±15.0	80.0±15.2	79.4±14.8	0.858
Heart Rate (beats/min)	80.8±18.2	80.9±18.4	80.8±18.1	0.659
Serum creatinine (µmol/l)[†]	80 (62-102)	81 (62-104)	80 (62-101)	0.309
NT-proBNP (pg/ml)[†]	1825 (462-5931)	1695 (408-5903)	1888 (556-5946)	0.760
LVEDD (mm)[†]	52 (46-62)	51 (46-63)	52 (46-61)	0.829
LAD (mm)[†]	38 (33-44)	38 (33-44)	39 (33-45)	0.990

Ejection fraction (%)†	56 (36-65)	56 (36-65)	56 (36-66)	0.961
Medication - (%)				
Digoxin	145 (15.8)	48 (14.8)	97 (16.3)	0.554
Diuretics	380 (41.3)	129 (39.8)	251 (42.2)	0.486
ACEI	518 (56.4)	175 (54.0)	343 (57.6)	0.288
ARB	74 (8.1)	31 (9.6)	43 (7.2)	0.213
Beta-blocker	432 (47.0)	153 (47.2)	279 (46.9)	0.923
Spironolactone	355 (39.9)	116 (37.4)	239 (41.3)	0.263

*For similarity among the different genotypes. §Listed as number (%). †Interquartile range included in parentheses. Abbreviations: ACEI, angiotensin-converting enzyme inhibitor; ARB, Angiotensin receptor blockers; LAD, left atrial dimension; LVEDD, left ventricular end-diastolic dimension; NT-proBNP, the N-terminal pro-hormone of brain natriuretic peptide; NYHA, New York Heart Association.

Table S3. Cox-Regression of the Clinical Factors Affecting the Primary End Point in the Entire Study Cohort

Variables	HR	95% CI	P value
Age	1.02	1.01 to 1.03	<0.001
Gender	1.05	0.85 to 1.28	0.671
Smoking	1.05	0.86 to 1.28	0.632
Hypertension	1.07	0.79 to 1.46	0.647
Hyperlipidemia	0.99	0.80 to 1.24	0.952
Diabetes	1.53	1.26 to 1.86	<0.001
Stroke	0.80	0.59 to 1.08	0.139
Etiology*	1.28	1.05 to 1.56	0.013
NYHA§	3.04	2.39 to 3.87	<0.001
NT-proBNP	1.55	1.45 to 1.67	<0.001

All of the data presented in this table are univariate Cox proportional hazards. *HR of etiology, calculated by comparing nonischemic versus ischemic HF. §HR of NYHA functional class, calculated by comparing class III + class IV versus class II HF. Abbreviations: CI, confidence interval; HF, heart failure; HR, hazard ratio; NT-pro-BNP, the N-terminal pro-hormone of brain natriuretic peptide; NYHA, New York Heart Association.

Table S4. Primary and Secondary End Points, According to Genotypes of Polymorphisms in the Pathway

Gene and Genotype	Primary End Point	All-cause Deaths	Cardiovascular Hospitalization	Recurrence of Heart Failure
GRK5 Gln41Leu A>T - no. (%)				
AA	411 (17.7)	437 (18.8)	696 (29.9)	611 (26.3)
AT	8 (13.6)	11 (18.6)	11 (18.6)	16 (27.1)
Log rank P value	0.303	0.812	0.029	0.71
GRK5 Arg304His G>A - no. (%)				
GG	233 (17.9)	255 (19.5)	379 (29.0)	323 (24.8)
GA	165 (17.7)	170 (18.2)	276 (29.6)	257 (27.5)
AA	21 (14.2)	23 (15.5)	53 (35.8)	48 (32.4)
Log rank P value	0.422	0.337	0.276	0.101
ADRB1Arg389Gly C>G - no. (%)				
CC	40 (19.0)	45 (21.4)	55 (26.2)	58 (27.6)
CG	176 (17.5)	185 (18.4)	304 (30.3)	270 (26.9)
GG	201 (17.3)	215 (18.5)	346 (29.7)	297 (25.5)
Log rank P value	0.973	0.793	0.349	0.679
ADRB2 Arg16Gly A>G - no. (%)				
AA	129 (14.0)	138 (15.0)	243 (26.4)	226 (24.6)
AG	209 (18.8)	226 (20.3)	355 (31.9)	304 (27.3)
GG	81 (22.8%)	84 (23.7)	110 (31.0)	98 (27.6)
Log rank P value	<0.001	<0.001	0.010	0.215
ADRB2 Gln27Glu C>G - no. (%)				
CC	332 (17.2)	358 (18.5)	574 (29.7)	517 (26.8)
CG	39 (22.2)	41 (23.3)	52 (29.5)	45 (25.6)
GG	48 (17.1)	49 (17.5)	82 (29.3)	66 (23.6)

Log rank P value	0.114	0.146	0.993	0.66

P values are two-sided and were calculated by a stratified log-rank test without adjustment for comparisons for the clinical events.

Table S5. Characteristics of Patients with Heart Failure Stratified by BB Use and *ADRB2* Arg16Gly A>G Genotype

Characteristics	All Patients (N=2386)		Patients with AA Genotype (N=919)		Patients with AG Genotype (N=1112)		Patients with GG Genotype (N=355)	
	BB Use (N=1365)	No BB Use (N=1021)	BB Use (N=534)	No BB Use (N=385)	BB Use (N=636)	No BB Use (N=476)	BB Use (N=195)	No BB Use (N=160)
Age (year)r	58.7±14.0*	60.1±15.2	58.6±14.1	59.0±14.9	58.8±13.9§	61.3±15.1	59.0±13.0	59.3±16.1
Male sex†	933 (68.4)*	654 (64.1)	363 (68.0)	249 (64.7)	446 (70.1)§	298 (62.6)	124 (63.6)§	107 (66.9)
Ischemic etiology†	708 (51.9)*	426 (41.7)	279 (52.2)§	171 (44.4)	330 (51.9)§	193 (40.5)	99 (50.8)	62 (38.8)
NYHA functional class†								
II	665 (48.7)	369 (36.1)	263 (49.3)	151 (39.2)	314 (49.5)	161 (40.5)	88 (45.1)	57 (35.6)
III	454 (33.3)*	353 (34.6)	182 (34.1)§	134 (34.8)	206 (32.4)§	169 (35.5)	66 (33.8)§	50 (31.3)
IV	246 (18.0)	299 (29.3)	89 (16.7)	100 (26.0)	116 (18.2)	146 (30.7)	41 (21.0)	53 (33.1)
Personal history†								
Smoking	554 (40.6)	374 (36.6)	218 (40.8)	145 (37.7)	265 (41.7)§	165 (34.7)	71 (36.4)	64 (40.0)
Hypertension	1192 (87.3)	896 (87.8)	454 (85.0)	322 (83.6)	566 (89.0)	430 (90.3)	71 (36.4)	64 (40.0)
Hyperlipidemia	368 (27.0)*	226 (22.1)	145 (27.2)	96 (24.9)	166 (26.1)§	96 (20.2)	57 (29.2)	34 (21.3)
Diabetes	419 (30.7)	340 (33.1)	165 (30.9)	125 (32.5)	185 (29.1)	163 (34.2)	69 (35.4)	52 (32.5)
Stroke	131 (9.6)	115 (11.3)	59 (11.0)	43 (11.2)	57 (9.0)	56 (11.8)	15 (7.7)	16 (10.0)
Clinical testing								
Systolic pressure (mmHg)	131.5±24.7	130.9±26.0	131.5±24.6	132.4±26.1	132.3±25.4	130.1±25.8	129±22.4	130±26.4
Diastolic pressure (mmHg)	80.7±16.0	80.0±16.4	80.7±16.7	80.9±16.9	81.1±15.7	79.8±16.8	79.2±15.0	77.8±13.7
Heart Rate (beats/min)	82.6±18.8	84.0±21.8	82.2±18.2	83.3±23.3	82.7±19.9	84.8±20.9	83.3±16.9	83.7±23.4

Serum Creatinine (µmol/l)‡	84 (67-102)*	84 (67-117)	82 (67-103)	83 (65-111)	84 (69-102)§	86 (69-119)	84 (66-101)	85 (66-122)
NT-proBNP (pg/ml)‡	1425 (307-4441)†	2417 (608-7586)	1582 (430-4114)§	2140 (547-6898)	1286 (255-4365)§	2516 (731-7906)	1576 (446-6427)	2795 (299-8392)
LVEDD (mm)‡	54 (47-64)	55 (49-64)	54 (47-64)	54 (48-63)	55 (48-64)	56 (49-64)	55 (47-63)	57 (49-66)
LAD (mm)‡	40 (35-45)	40 (35-46)	39 (35-45)	39 (34-45)	39 (35-45)§	41 (36-46)	40 (36-45)	41 (36-46)
Ejection fraction (%)‡	47 (32-62)	47 (33-60)	47 (32-61)	50 (35-61)	50 (32-62)§	45 (32-59)	44 (32-63)	44 (33-60)
Medication†								
Digoxin	351 (25.7)*	212 (20.8)	148 (27.7)§	66 (17.1)	160 (25.2)	114 (23.9)	43 (22.1)	32 (20.0)
Diuretics	679 (49.7)*	425 (41.6)	262 (49.1)§	140 (36.4)	314 (49.4)	213 (44.7)	103 (52.8)	72 (45.0)
ACEI	858 (62.9)*	388 (38.0)	320 (59.9)§	146 (37.9)	405 (63.7)§	179 (37.6)	133 (68.2)§	63 (39.4)
ARB	204 (14.9)*	75 (7.3)	77 (14.4)§	25 (6.5)	105 (16.5)§	37 (7.8)	22 (11.3)	13 (8.1)
Spironolactone	594 (43.5)*	344 (33.7)	238 (44.6)§	116 (30.1)	267 (42.0)§	170 (35.7)	89 (45.6)	58 (36.3)

*P value <0.05 versus all patients not using BBs. §P value<0.05 versus patients the same genotype who are not using BBs. †Listed as number (%). ‡Interquartile range included in parentheses.

Abbreviations: ACEI, angiotensin-converting enzyme inhibitor; ARB, Angiotensin receptor blockers; BB, β-blocker; LAD, left atrial dimension; LVEDD, left ventricular end-diastolic dimension; NT-proBNP, the N-terminal pro-hormone of brain natriuretic peptide; NYHA, = New York Heart Association.

Table S6. Characteristics of Heart Failure Subjects by β-blocker Use and *ADRB1* Arg389Gly C>G Genotype

Characteristics	GG Genotype (N=210)		CG Genotype (N=1003)		CC Genotype (N=1164)	
	BB Use (N=123)	No BB Use (N=87)	BB Use (N=573)	No BB Use (N=430)	BB Use (N=666)	No BB Use (N=498)
Age - yr	58.1±14.4	59.0±16.3	58.3±13.9	59.8±15.4	59.2±13.7	60.6±14.9
Male sex - (%)	89 (72.4)	58 (66.7)	402 (70.2)[※]	264 (61.4)	439 (65.9)	328 (65.9)
Ischemic etiology - (%)	61 (49.6)	33 (37.9)	268 (46.8)[※]	169 (39.3)	376 (56.5)[※]	220 (44.2)
NYHA functional class - (%)						
Ⅱ	45 (36.6)	28 (32.2)	276 (48.2)	158 (36.7)	343 (51.5)	180 (36.1)
Ⅲ	49 (39.8)[※]	24 (27.6)	198 (34.6)[※]	150 (34.9)	205 (30.8)[※]	177 (35.5)
Ⅳ	29 (23.6)	35 (40.2)	99 (17.3)	122 (28.4)	118 (17.7)	141 (12.1)
Personal history - (%)						
Smoking	51 (41.5)	26 (29.9)	235 (41.0)[※]	147 (34.2)	267 (40.1)	198 (12.0)
Hypertension	106 (86.2)	74 (85.1)	506 (88.3)	383 (89.1)	577 (86.6)	434 (87.1)
Hyperlipidemia	37 (30.1)	23 (26.4)	154 (26.9)	97 (22.6)	176 (26.4)[※]	104 (20.9)
Diabetes	41 (33.3)	25 (28.7)	182 (31.8)	135 (31.4)	195 (29.3)[※]	178 (35.7)
Stroke	7 (5.7)	9 (10.3)	49 (8.6)	45 (10.5)	75 (11.3)	60 (12.0)
Clinical testing						
Systolic pressure - mm Hg	131.3±23.9	131.6±26.8	131.8±24.9	129.6±25.2	131.2±24.7	132.0±26.5
Diastolic pressure - mm Hg	81.5±16.2	80.3±15.9	81.0±16.7	80.0±16.9	80.2±15.3	80.0±16.2
Heart Rate - beats/min	84.6±18.3	83.8±15.9	82.3±19.1	83.5±21.7	82.5±18.8	84.6±21.7
Serum Creatinine - umol/L	86 (72-107)	83 (67-113)	84 (68-99)	83 (67-113)	83 (67-103)[※]	85 (67-121)

NT-proBNP - pg/mL	2458 (688-4780)	2465 (706-9000)	1510 (334-4615)※	2556 (658-7797)	1204 (253-3968)※	2400 (498-7298)
LVEDD - mm	56 (47-66)	56 (48-67)	55 (48-65)	56 (49-64)	54 (47-62)※	55 (49-63)
LAD - mm	41 (35-47)	42 (33-47)	40 (35-45)	40 (35-46)	39 (34-45)	40 (35-45)
Ejection fraction - (%)	44 (32-58)	47 (35-60)	45 (32-60)	46 (33-60)	50 (33-63)	47 (33-60)
Medication - (%)						
Digoxin	32 (26.0)	14 (16.1)	164 (28.6)※	91 (21.2)	155 (23.3)	107 (21.5)
Diuretics	65 (52.8)	41 (47.1)	302 (52.7)※	177 (41.2)	310 (46.5)	205 (41.2)
ACEI	80 (65.0)※	35 (40.2)	360 (62.8)※	155 (36.0)	417 (62.6)※	197 (39.6)
ARB	17 (13.8)	8 (9.2)	78 (13.6)※	27 (6.3)	109 (16.4)※	37 (7.4)
Spironolactone	57 (46.3)	35 (40.2)	268 (46.8)※	147 (34.2)	268 (40.2)※	161 (32.3)

NYHA = New York Heart Association; NT-proBNP = The N-terminal prohormone of brain natriuretic peptide; LVEDD =left ventricular end-diastolic dimension; LAD = left atrial dimension; BB = beta-blocker; ACEI = angiotensin-converting enzyme inhibitor; ARB = angiotensin receptor blocker

※ P value<0.05 versus patients with no BB use of the same genotype

Table S7. Characteristics of Heart Failure Subjects by β-blocker Use and *ADRB2* Gln27Glu C>G Genotype

	CC Genotype (N=1930)		CG Genotype (N=176)		GG Genotype (N=280)	
**Characteristics**	**BB Use (N=1096)**	**No BB Use (N=834)**	**BB Use (N=105)**	**No BB Use (N=71)**	**BB Use (N=164)**	**No BB Use (N=116)**
Age - yr	58.8±13.8※	60.1±15.0	58.5±13.4	59.4±16.7	58.8±14.3	60.5±16.1
Male sex - (%)	749 (68.3)※	527 (63.2)	71 (67.6)	54 (76.1)	113 (68.9)	73 (62.9)
Ischemic etiology - (%)	571 (52.1)※	346 (41.5)	53 (50.5)	29 (40.8)	84 (51.2)	51 (44.0)
NYHA functional class - (%)						
Ⅱ	542 (49.5)	293 (35.1)	41 (39.0)	27 (38.0)	82 (50.0)	49 (42.2)
Ⅲ	361 (32.9)※	301 (36.1)	43 (41.0)	23 (32.4)	50 (30.5)※	29 (25.0)
Ⅳ	193 (17.6)	240 (28.8)	21 (20.0)	21 (29.6)	32 (19.5)	38 (32.8)
Personal history - (%)						
Smoking	451 (41.1)※	302 (36.2)	40 (38.1)	32 (45.1)	63 (38.4)	40 (34.5)
Hypertension	958 (87.4)	732 (87.8)	88 (83.8)	61 (85.9)	146 (89.0)	103 (88.8)
Hyperlipidemia	283 (25.8)	191 (22.9)	33 (31.4)	14 (19.7)	52 (31.7)※	21 (18.1)
Diabetes	324 (29.6)※	284 (34.1)	37 (35.2)	18 (25.4)	58 (35.4)	38 (32.8)
Stroke	107 (9.8)	89 (10.7)	7 (6.7)	11 (15.5)	17 (10.4)	15 (12.9)
Clinical testing						
Systolic pressure - mm Hg	131.7±24.9	130.9±25.9	128.7±23.1	130.8±22.1	132.4±24.5	130.8±29.0
Diastolic pressure - mm Hg	80.7±16.1	79.7±15.8	79.6±15.5	81.2±14.9	80.7±15.3	80.7±20.9
Heart Rate - beats/min	82.8±19.1	83.6±21.5	82.5±17.6	87.2±20.3	81.3±17.8	85.6±24.7
Serum Creatinine - umol/L	83 (67-101)	83 (66-114)	83 (65-103)	91 (68-135)	87 (70-104)	92 (76-128)

NT-proBNP - pg/mL	1386 (307-4389)※	2359 (590-7109)	1997 (575-4894)	2719 (500-9203)	1186 (255-4567)※	2764 (741-8897)
LVEDD - mm	54 (47-64)	55 (48-63)	55 (48-65)	58 (51-67)	55 (47-63)	57 (48-64)
LAD - mm	39 (35-45)	40 (35-45)	41 (37-46)	41 (35-47)	39 (34-45)	41 (35-48)
Ejection fraction - (%)	48 (32-62)	47 (33-60)	40 (31-60)	42 (30-60)	49 (33-62)	48 (33-62)
Medication - (%)						
Digoxin	280 (25.5)※	172 (20.6)	26 (24.8)	12 (16.9)	45 (27.4)	28 (24.1)
Diuretics	538 (49.1)※	337 (40.4)	60 (57.1)	30 (42.3)	81 (49.4)	58 (50.0)
ACEI	682 (62.2)※	318 (38.1)	65 (61.9)※	29 (40.8)	111 (67.7)※	41 (35.3)
ARB	162 (14.8)※	55 (6.6)	13 (12.4)	4 (5.6)	29 (17.7)	16 (13.8)
Spironolactone	476 (43.4)※	278 (33.3)	52 (49.5)※	23 (32.4)	66 (40.2)	43 (37.1)

NYHA = New York Heart Association; NT-proBNP = The N-terminal prohormone of brain natriuretic peptide; LVEDD =left ventricular end-diastolic dimension; LAD = left atrial dimension; BB = beta-blocker; ACEI = angiotensin-converting enzyme inhibitor; ARB = angiotensin receptor blocker;

※ P value<0.05 versus patients with no BB use of the same genotype

Table S8. Characteristics of Heart Failure Subjects by β-blocker Use and *GRK5* Gln41Leu A>T Genotype

	AA Genotype (N=2325)		AT Genotype (N=59)	
	**BB Use**	**No BB Use**	**BB Use**	**No BB Use**
**Characteristics**	**(N=1327)**	**(N=998)**	**(N=38)**	**(N=21)**
Age - yr	58.8±13.8[※]	60.0±15.3	58.3±14.3	65.1±12.5
Male sex - (%)	907 (68.3)[※]	642 (64.3)	26 (68.4)	12 (57.1)
Ischemic etiology - (%)	692 (52.1)[※]	415 (41.6)	16 (42.1)	10 (47.6)
NYHA functional class - (%)				
Ⅱ	647 (48.8)	362 (36.3)	18 (47.4)	7 (33.3)
Ⅲ	444 (33.5)[※]	349 (35.0)	10 (26.3)	3 (14.3)
Ⅳ	236 (17.8)	287 (28.8)	10 (26.3)	11 (52.4)
Personal history - (%)				
Smoking	540 (40.7)	370 (37.1)	14 (36.8)	4 (19.0)
Hypertension	1160 (87.4)	874 (87.6)	32 (84.20	20 (95.2)
Hyperlipidemia	358 (27.0)[※]	224 (22.4)	10 (26.3)	1 (4.8)
Diabetes	410 (30.9)	331 (33.2)	9 (23.7)	8 (38.1)
Stroke	128 (9.6)	112 (11.2)	3 (7.9)	3 (14.3)
Clinical testing				
Systolic pressure - mm Hg	131.3±24.6	130.8±26.1	138.4±28.0	132.3±23.5
Diastolic pressure - mm Hg	80.5±15.9	80.0±16.5	86.1±18.7	79.4±11.4
Heart Rate - beats/min	82.5±15.9	83.9±21.8	87.2±15.5	88.1±23.8
Serum Creatinine - umol/L	84 (67-101)[※]	84 (67-116)	84 (73-143)	112 (73-269)

NT-proBNP - pg/mL	1428 (306-4458)※	2402 (597-7668)	1360 (560-4389)	8630 (853-34696)
LVEDD - mm	54 (47-64)	55 (49-64)	56 (47-65)	57 (49-61)
LAD - mm	39 (35-45)	40 (35-46)	42 (36-48)	38 (33-43)
Ejection fraction - (%)	47 (32-62)※	47 (33-60)	38 (31-62)	43 (35-57)
Medication - (%)				
Digoxin	341 (25.7)※	208 (20.8)	10 (26.3)	4 (19.0)
Diuretics	658 (49.6)※	414 (41.5)	21 (55.3)	10 (47.6)
ACEI	829 (62.5)※	498 (37.5)	29 (76.3)※	7 (33.3)
ARB	198 (14.9)※	73 (7.3)	6 (15.8)	2 (9.5)
Spironolactone	576 (43.4)※	336 (33.7)	18 (47.4)	8 (38.1)

NYHA = New York Heart Association; NT-proBNP = The N-terminal prohormone of brain natriuretic peptide; LVEDD =left ventricular end-diastolic dimension; LAD = left atrial dimension; BB = beta-blocker; ACEI = angiotensin-converting enzyme inhibitor; ARB = angiotensin receptor blocker;

※ P value<0.05 versus patients with no BB use of the same genotype

Table S9. Characteristics of Heart Failure Subjects by β-blocker Use and *GRK5* Arg304His G>A Genotype

Characteristics	GG Genotype (N=1305)		GA Genotype (N=933)		AA Genotype (N=148)	
	BB Use (N=725)	No BB Use (N=580)	BB Use (N=553)	No BB Use (N=380)	BB Use (N=87)	No BB Use (N=61)
Age - yr	58.9±13.6	60.5±15.6	58.5±13.8	59.7±14.8	58.4±16.3	58.7±14.3
Male sex - (%)	506 (69.8)[※]	369 (63.6)	374 (67.6)	237 (62.4)	53 (60.9)[※]	48 (78.7)
Ischemic etiology - (%)	373 (51.4)[※]	239 (41.2)	285 (51.5)[※]	162 (42.6)	50 (57.5)[※]	25 (41.0)
NYHA functional class - (%)						
Ⅱ	360 (49.7)	207 (35.7)	262 (47.4)	144 (37.9)	43 (49.4)	18 (29.5)
Ⅲ	236 (32.6)[※]	201 (34.7)	189 (34.2)[※]	127 (33.4)	29 (33.3)[※]	25 (41.0)
Ⅳ	129 (17.8)	172 (29.7)	102 (18.4)	109 (28.7)	15 (17.2)	18 (29.5)
Personal history - (%)						
Smoking	295 (40.7)	209 (36.0)	225 (40.7)	139 (36.6)	34 (39.1)	26 (42.6)
Hypertension	625 (86.2)	498 (85.9)	489 (88.4)	347 (91.3)	133.4±25.4	134.6±26.8
Hyperlipidemia	198 (27.3)[※]	130 (22.4)	142 (25.7)	84 (22.1)	28 (32.2)	12 (19.7)
Diabetes	210 (29.0)	192 (33.1)	176 (31.8)	124 (32.6)	33 (37.9)	24 (39.3)
Stroke	78 (10.8)	70 (12.1)	41 (7.4)	37 (9.7)	12 (13.8)	8 (13.1)
Clinical testing						
Systolic pressure - mm Hg	131.8±25.0	130.0±22.4	130.8±24.2	131.6±24.9	133.4±25.4	134.6±26.8
Diastolic pressure - mm Hg	81.0±16.6	79.3±16.1	80.3±15.5	80.3±15.9	80.3±13.7	83.2±21.8
Heart Rate - beats/min	82.6±18.8	83.9±21.8	82.9±19.2	84.2±21.6	81.4±16.8	84.7±23.2
Serum Creatinine - umol/L	84 (67-102)	83 (67-120)	83 (68-103)	86 (67-114)	84 (64-101)	85 (68-109)

NT-proBNP - pg/mL	1660 (325-4564)※	2629 (659-7668)	1315 (307-4387)※	2257 (558-7259)	1114 (280-4032)	1531 (617-8925)
LVEDD - mm	55 (47-64)	55 (48-64)	54 (47-63)	56 (48-64)	53 (47-61)	55 (49-63)
LAD - mm	39 (35-45)	40 (35-46)	40 (35-45)	40 (34-45)	39 (36-45)	42 (35-46)
Ejection fraction - (%)	47 (31-61)	47 (33-60)	50 (33-62)	45 (32-60)	54 (38-63)	54 (35-61)
Medication - (%)						
Digoxin	199 (27.4)※	115 (19.8)	130 (23.5)	83 (21.8)	22 (25.3)	14 (23.0)
Diuretics	367 (50.6)※	240 (41.4)	268 (48.5)※	157 (41.3)	44 (50.6)	28 (45.9)
ACEI	473 (65.2)※	205 (35.3)	333 (60.2)※	155 (40.8)	52 (59.8)	28 (45.9)
ARB	95 (13.1)※	46 (7.9)	15 (17.2)※	2 (3.3)	15 (17.2)※	2 (3.3)
Spironolactone	318 (43.9)※	192 (33.1)	41 (47.1)※	18 (29.5)	41 (47.1)※	18 (29.5)

NYHA = New York Heart Association; NT-proBNP = The N-terminal prohormone of brain natriuretic peptide; LVEDD =left ventricular end-diastolic dimension; LAD = left atrial dimension; BB = beta-blocker; ACEI = angiotensin-converting enzyme inhibitor; ARB = angiotensin receptor blocker;

※ P value<0.05 versus patients with no BB use of the same genotype

Table S10. Characteristics of Heart Failure Subjects by β-blocker Use and *ADRB2* Genotype Groups

Characteristics	Group A (N=883)		Group B (N=1078)		Group C (N=425)	
	BB Use (N=513)	No BB Use (N=370)	BB Use (N=603)	No BB Use (N=475)	BB Use (N=249)	No BB Use (N=176)
Age - yr	58.6±14.1	59.0±14.9	59.0±13.6[※]	61.0±15.0	58.5±13.9	60.0±16.4
Male sex - (%)	348 (67.8)	240 (64.9)	415 (68.8)[※]	295 (62.1)	170 (68.3)	119 (67.6)
Ischemic etiology - (%)	269 (52.4)[※]	166 (44.9)	311 (51.6)[※]	185 (38.9)	128 (51.4)	75 (42.6)
NYHA functional class - (%)						
Ⅱ	257 (50.1)	144 (38.9)	290 (48.1)	155 (32.6)	118 (47.4)	70 (39.8)
Ⅲ	173 (33.7)[※]	130 (35.1)	197 (32.7)[※]	174 (36.6)	84 (33.7)[※]	49 (27.8)
Ⅳ	83 (16.2)	96 (25.9)	116 (19.2)	146 (30.7)	47 (18.9)	57 (32.4)
Personal history - (%)						
Smoking	209 (40.7)	139 (37.6)	250 (41.5)[※]	168 (35.4)	95 (38.2)	67 (38.1)
Hypertension	439 (85.6)	309 (83.5)	534 (88.6)	432 (90.9)	219 (88.0)	155 (88.1)
Hyperlipidemia	140 (27.3)	93 (25.1)	148 (24.5)	101 (21.3)	80 (32.1)[※]	32 (18.2)
Diabetes	160 (31.2)	123 (33.2)	168 (27.9)[※]	161 (33.9)	91 (36.5)	56 (31.8)
Stroke	57 (11.1)	42 (11.4)	52 (8.6)	48 (10.1)	22 (8.8)	25 (14.2)
Clinical testing						
Systolic pressure - mm Hg	131.8±24.8	132.6±26.3	131.4±24.8	129.6±25.3	131.2±24.3	130.8±26.9
Diastolic pressure - mm Hg	80.7±16.8	80.8±17.0	80.8±15.4[※]	78.8±14.8	80.2±15.5	80.9±19.1
Heart Rate - beats/min	82.3±18.3	83.0±22.4	83.3±19.7	84.0±20.6	81.7±17.8[※]	86.3±23.6
Serum Creatinine - umol/L	82 (67-103)	83 (65-111)	83 (68-100)	82 (67-115)	87 (68-104)[※]	94 (75-133)

NT-proBNP - pg/mL	1505 (362-4127)※	2107 (544-6846)	1419 (310-4578)※	2450 (639-7378)	1367 (292-4708)※	2917 (702-9000)
LVEDD - mm	53 (47-64)	54 (48-63)	55 (47-64)	56 (49-64)	55 (47-63)	58 (49-66)
LAD - mm	39 (35-45)	39 (34-45)	40 (35-45)	40 (36-46)	40 (35-45)	41 (35-48)
Ejection fraction - (%)	47 (32-62)	50 (35-61)	48 (32-62)	44 (32-58)	47 (33-62)	47 (32-60)
Medication - (%)						
Digoxin	139 (27.1)※	62 (16.8)	149 (24.7)	111 (23.4)	63 (25.3)	39 (22.2)
Diuretics	249 (48.5)※	134 (36.2)	301 (49.9)※	206 (43.4)	129 (51.8)	85 (48.3)
ACEI	309 (60.2)※	140 (37.8)	384 (63.7)※	181 (38.1)	165 (66.3)※	67 (38.1)
ARB	74 (14.4)※	25 (6.8)	91 (15.1)※	30 (6.3)	39 (15.7)	20 (11.4)
Spironolactone	226 (44.1)※	110 (29.7)	261 (43.3)※	170 (35.8)	107 (43.0)	64 (36.4)

NYHA = New York Heart Association; NT-proBNP = The N-terminal prohormone of brain natriuretic peptide; LVEDD =left ventricular end-diastolic dimension; LAD = left atrial dimension; BB = beta-blocker; ACEI = angiotensin-converting enzyme inhibitor; ARB = angiotensin receptor blocker

※ P value<0.05 versus patients with no BB use of the same genotype

Table S11. Primary Endpoint According to *ADRB2* Arg16Gly (A>G) and β-Blocker Treatment in Validation Cohort

Outcome[#]	AA Genotype (N=324)	AG/GG Genotype (N=595)	HR[§] (95% CI) AG/GG VS. AA	P value
**BB Use**	7 (4.6)	14 (5.0)	1.07 (0.43-2.65)	0.883
**No BB Use**	11 (6.4)	36 (12.4)	2.09 (1.06-4.12)	0.034
**HR[※] (95% CI)**	0. 86 (0.31-2.42)	0. 49 (0.26-0.92)	/	/
**P value**	0.774	0.026	/	/

[#] Outcome were presented as counts (percentage)

[§] HR, Hazard Ratios and P value were calculated with the use of stratified Cox proportional-hazard models adjusting with gender, age, and New York Heart Association functional class.

[※] HR, Hazard Ratios and P value were calculated with the use of stratified Cox proportional-hazard models adjusting with gender, age, and New York Heart Association functional class and other drug treatments.

Table S12. The Primer Sequences and Size of Amplicons in the First Re-sequencing Stage

Gene/Exon	Sense primer	Anti-sense primer	Amplicon size (bp)
ADRB1			
E1	5' CGGGCTTCTGGGGTGTTC 3'	5' GCGTCAGCAGGCTCTGGTAG 3'	587
E2	5' CCAGCGAAAGCCCCGAG 3'	5' AACACCCGCAGGTACACGAA 3'	607
E3	5' GCCCTGGTGTCCTTCCTGC 3'	5' GCGTAGCCCAGCCAGTTGA 3'	545
E4	5' GCCTCGTCCGTAGTCTCCTTC 3'	5' CACAGATCTCCTCGTTCCCCT 3'	815
E5	5' GCCTTCAACCCCATCATCTACTG 3'	5' CTCCCATCCCTTCCCAAACTT 3'	502
ADRB2			
E1	5' GAATGAGGCTTCCAGGCGTC 3'	5' GGCATAGGCTTGGTTCGTGA 3'	703
E2	5' CGGTCACCAACTACTTCATCACTTC 3'	5' CATCCTGCTCCACCTGGCTAA 3'	558
E3	5' CTTACCTCCTTCTTGCCCATTCA 3'	5' CCCTGTGTTGCCGTTGCTG 3'	597
E4	5' ATTCTGGTTTCAATCCCCTTAT 3'	5' AAATAGTCTGTTTAGTGTTCTGTTGG 3'	348
GRK2			
E1	5' GCCGACGTGAGCTACCTGATG 3'	5' GGGACCGATGGGGCAGAG 3'	335
E2	5' CACCACCACAGTGCCCTCAG 3'	5' CAAAAACCCCAAAGGAGGAACT 3'	434
E3	5' GCCACCTCCATAGCCAGTTCT 3'	5' CTCCGTCTCCAGCTTCTCGTAC 3'	357
E4	5' GGGGCGGCTTTGTGGAAC 3'	5' CCCGAGAGACCGTGGTTGC 3'	480
E5	5' GGAGGAGGAGCGTGTGGC 3'	5' CCCCGAGAGACCGTGGTT 3'	363
E6	5' GAAGAGAGGACCCTGCTGAGAAT 3'	5' AGCCCACGGGAGAGAAAGAG 3'	612
E7	5' CAAGATTGTGCCACAGCCCT 3'	5' AAATGAACTGTTTCCCACCGC 3'	441
E8	5' AGGTAAATATGTGGCAAGGATGG 3'	5' CAGCACGGCAAGACAGGAGT 3'	350
E9	5' ACACGCTGGTCCTGGGTCTA 3'	5' GGGGCGGGCAGTTAGC 3'	271
E10	5' GCCGCTGAGGCTCTGGA 3'	5' CTCTGAGAAGACCCCGTGCT 3'	289

E11	5'	TTCCACACGCCAGACAAGC	3'	5'	AGAGGACAGCCACGGAGGT	3'	317
E12	5'	CCCACCTTTGCCCTTTCTTTG	3'	5'	CGTGGGTGCCCCTGTGG	3'	300
E13	5'	CGTGTGAGTGCCCCCCA	3'	5'	ACTTCTCTTAGCCCCAACCCA	3'	228
E14	5'	GGCACCGTCCCTGACTTTG	3'	5'	GGCTGGTGGGAAGCAAGAGT	3'	251
E15	5'	CGCCCTCCCCGTGCT	3'	5'	CTGGCCATGCCCCAACTTA	3'	389
E16	5'	GCCCAAGTTCACAGAGCCAGA	3'	5'	TCGTGGGGGGATCAGCG	3'	268
E17	5'	CCCTGGACTGGCAGATGG	3'	5'	GCTGGCACAGGGTCTCACA	3'	305
E18	5'	TTGCACTGACCATCCCTACCC	3'	5'	GGTGAGTTCCTTAAACCCGAGC	3'	351
E19	5'	CAAGATTGTGCCACAGCCCT	3'	5'	AAATGAACTGTTTCCCACCGC	3'	441
E20	5'	CAGTGGCAGCGGCGG	3'	5'	GCCCCTCGGCCCTCC	3'	314
E21	5'	CGATGTGAGTGGGGGCTGA	3'	5'	GGGAAAACAAATAATAAAAATTCGACA	3'	330
GRK5							
E1	5'	GAGGAGAATGGAGTGACAGAGACA	3'	5'	CATCCAAATCCCTGCAACCT	3'	580
E2	5'	GTCTTGGGCTGGGGGTGT	3'	5'	ACTGTGTCCACTGATTGGCATT	3'	373
E3	5'	TGAGAGTTTGCCAGTCACCTTC	3'	5'	CTCCCATAATAACCCAGGCTTC	3'	528
E4	5'	TTTGGACTAAACATCGGGGC	3'	5'	CAGGAGGTCAACAACAGCCAC	3'	386
E5	5'	GGAGCGTGGCTGGTTCTTG	3'	5'	TCTTTGGACTTGGCACCTTGAT	3'	384
E6	5'	ATGCCAACCCCTTGCTCC	3'	5'	AAAATGTGTACCCTCTGCCTCC	3'	402
E7	5'	GTCCAGTCTCCAGCGATGATTC	3'	5'	CCACTGACTCTCTCCCACCCT	3'	379
E8	5'	TTATCTAAGCCTTGGTGTGAGTGG	3'	5'	CGTGTGATGGAGGAATGTCTTG	3'	586
E9	5'	ACGCCATTCTGTTGGATTCAC	3'	5'	CCCACCCCCTCGGACTTA	3'	519
E10	5'	CAAACCAGACAGCCTGAGCC	3'	5'	ACAAACAAGATGAGAGGTGGAGAAC	3'	555
E11	5'	GGACAGATGAGAATGCCGAGA	3'	5'	CCAGACCCCTGCTTCAATGT	3'	524

E12	5'	AGGAGAGTCATCAGGGCAAGC	3'	5'	TGTTCCCCTCACCCCTCTTC	3'	620
E13	5'	CCCAGGAACGAGGTCTGTAATC	3'	5'	ACAGGGAGGAAACTGACGCTT	3'	679
E14	5'	GGTCGCACAAAAAAACCACAG	3'	5'	CCTCCTTCCACCCGTCAGTC	3'	448
E15	5'	ATGAAGGCAGCACACAAAAGC	3'	5'	GGCGGAGAAACCCAGAGG	3'	586
E16	5'	TGCTCTTCTCCCGTGTTACAGG	3'	5'	AGGGGCTCCACTACTTCCACTT	3'	543

Table S13. The Sequences of Primers and Taqman Probes for Genotyping

Gene	Polymorphism		sequences
ADRB2	Arg16Gly		
		forward primer	5' GGCAGCGCCTTCTTGCT 3'
		reverse primer	5' AGGACGATGAGAGACATGACGA 3'
		probe1	FAM- ACCCAATAGAAGCCA –MGB
		probe2	HEX- ACCCAATGGAAGCC -MGB
ADRB2	Gln27Glu		
		forward primer	5' GCCCACCACCCACACCT 3'
		reverse primer	5' TTCTTGCTGGCACCCAATG 3'
		probe1	FAM- TCCCTTTGCTGCGTGA –MGB
		probe2	HEX- TCCCTTTCCTGCGTGA-MGB
GRK5	Gln41Leu		
		forward primer	5' CAGCTTACCTATGGTCCTTCGG 3'
		reverse primer	5' AGGCATCTTTTTCCCCATCTCT 3'
		probe1	FAM- CTTCACACTGGCTAAT –MGB
		probe2	HEX- CTTCACACAGGCTAAT-MGB
GRK5	Arg304His		
		forward primer	5' AGATCCTCTGCGGCTTAGAAGA 3'
		reverse primer	5' AGGACGATGAGAGACATGACGA 3'
		probe1	FAM- ACCCAATAGAAGCCA –MGB
		probe2	HEX- ACCCAATGGAAGCC -MGB

ADRB1 Arg389Gly C>G was synthesize by Invitrogen Company and the product ID is 8898494_10

Table S14. The Basic Characteristics of the Patients Enrolled for the Lymphocytes Experiments

	Genotype of ADRB2 Arg16Gly A>G			
	AA	AG	GG	
Characteristic	(n=10)	(n=10)	(n=6)	P Value
age - yr	56.9±13.5	69.2±11.7	56.2±8.9	0.050
Male sex - (%)	9 (90)	7 (70)	4 (66.7)	0.452
Ischemic etiology - (%)	2 (20)	5 (50)	5 (83.3)	0.046
NYHA functional class - (%)				
II	4 (40)	4 (40)	2 (33.3)	
III	3 (30)	4 (40)	2 (33.3)	0.973
IV	3 (30)	2 (20)	2 (33.3)	
Personal history - (%)				
Smoking	6 (60)	0 (0)	3 (50)	0.012
Hypertension	10 (100)	9 (90)	4 (66.7)	0.127
Hyperlipidemia	3 (30)	3 (30)	1 (16.7)	0.812
Diabetes	3 (30)	4 (40)	3 (50)	0.723
Stroke	0 (0)	1 (10)	0 (0)	0.435
Clinical testing				
Systolic pressure - mm Hg	128.8±22.8	122.2±27.2	120.0±19.3	0.435
Diastolic pressure - mm Hg	85.6±15.9	88.0±29.2	81.5±5.5	0.836
Heart Rate - beats/min	80.8±15.7	87.3±31.8	86.3±17.9	0.813
Serum Creatinine - umol/L	80 (72-90)	89 (64-116)	78 (48-106)	0.880
NT-proBNP - pg/mL	1613 (444-6410)	1860 (1268-4335)	4919 (272-7827)	0.860
LVEDD - mm	60 (51-63)	57 (51-67)	72 (68-77)	0.017

LAD - mm	46 (37-50)	37 (33-48)	45 (39-52)	0.674
Ejection fraction - %	34 (26-44)	38 (33-51)	29 (23-40)	0.100
Medication				
Digoxin	5 (50)	3 (30)	4 (66.7)	0.346
Diuretics	6 (60)	6 (60)	4 (66.7)	0.958
ACEI	6 (60)	7 (70)	5 (83.3)	0.618
Beta-blocker	4 (40)	8 (80)	5 (88.3)	0.098
Spironolactone	5 (50)	5 (50)	3 (50)	1.000

NYHA = New York Heart Association; NT-proBNP = The N-terminal prohormone of brain natriuretic peptide;

LVEDD =left ventricular end-diastolic dimension; LAD = left atrial dimension; ACEI = angiotensin-converting enzyme inhibitor.

Fig. S1

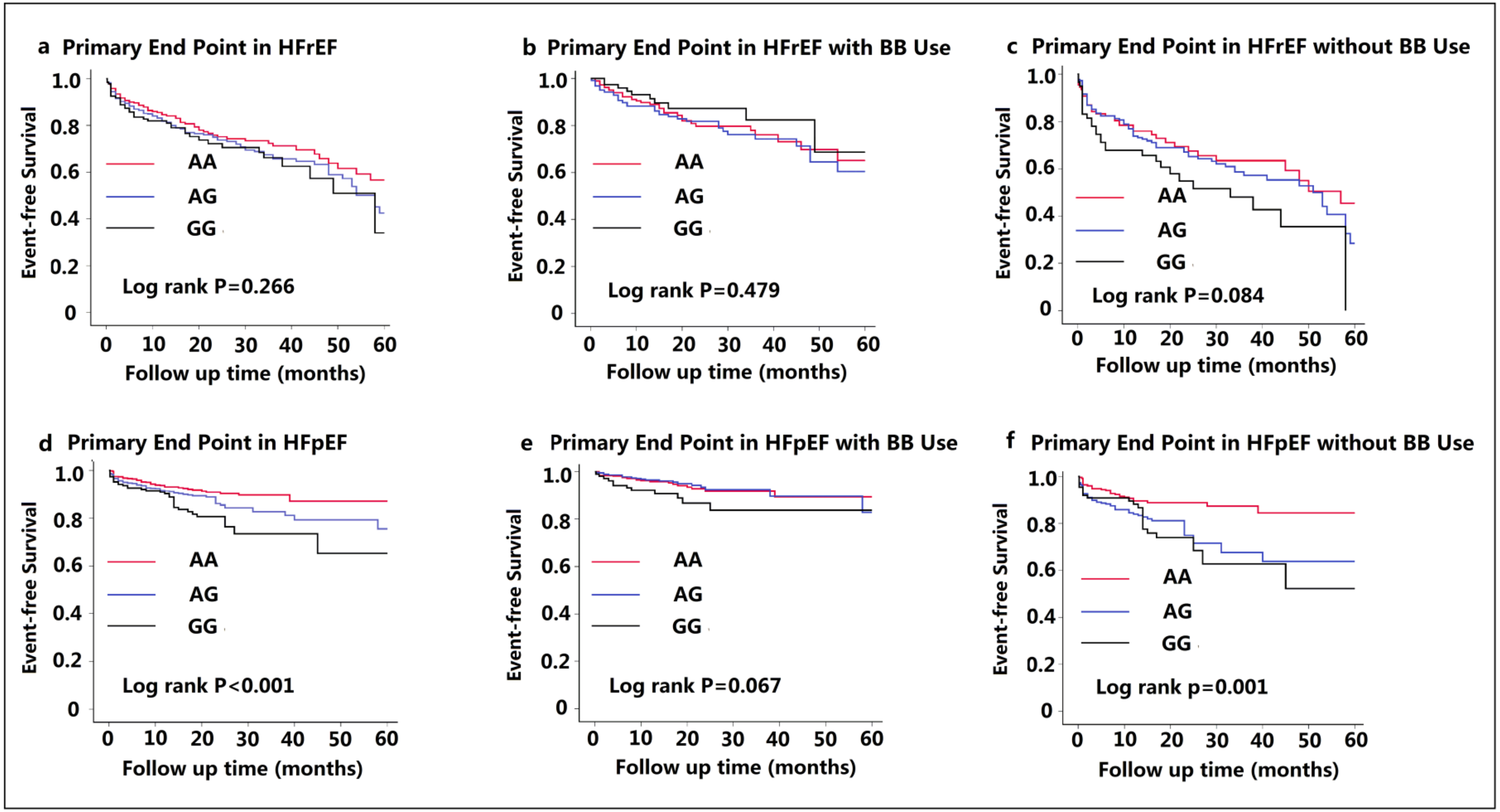
a Primary End Point in HFrEF
Event-free Survival
1.0
0.8
0.6
0.4
0.2
0
AA
AG
GG
Log rank P=0.266
0 10 20 30 40 50 60
Follow up time (months)
b Primary End Point in HFrEF with BB Use
Event-free Survival
1.0
0.8
0.6
0.4
0.2
0
AA
AG
GG
Log rank P=0.479
0 10 20 30 40 50 60
Follow up time (months)
c Primary End Point in HFrEF without BB Use
Event-free Survival
1.0
0.8
0.6
0.4
0.2
0
AA
AG
GG
Log rank P=0.084
0 10 20 30 40 50 60
Follow up time (months)
d Primary End Point in HFpEF
Event-free Survival
1.0
0.8
0.6
0.4
0.2
0
AA
AG
GG
Log rank P<0.001
0 10 20 30 40 50 60
Follow up time (months)
e Primary End Point in HFpEF with BB Use
Event-free Survival
1.0
0.8
0.6
0.4
0.2
0
AA
AG
GG
Log rank P=0.067
0 10 20 30 40 50 60
Follow up time (months)
f Primary End Point in HFpEF without BB Use
Event-free Survival
1.0
0.8
0.6
0.4
0.2
0
AA
AG
GG
Log rank p=0.001
0 10 20 30 40 50 60
Follow up time (months)

Fig. S1. The Primary Endpoint in Subgroups Stratified by Left Ventricular Ejection Fraction

Kaplan-Meier curves demonstrating that the primary composite end point did not varied among the groups of patients with the different *ADRB2* Arg16Gly genotype in the subgroup of patients with reduced ejection fraction (EF<40%, that is HFrEF) with log rank p=0.266 (a). The genotype of *ADRB2* Arg16Gly site did not predict the risk of primary end point for the patients with (b) or without (c) the treatment of β-blockers (Log rank p=0.479 and 0.084 respectively). In the subgroup of patients with preserved ejection fraction (EF≥40%, that is HFpEF), the primary endpoint significantly varied in different genotype groups with log rank p<0.001 (d). The genotype dependent variability in prognosis was still significant in the subgroup of patients without β-blockers (log rank p=0.001, f), while disappeared in the subgroup of patients with β-blocker treatment (log rank p=0.067, e).

Fig. S2

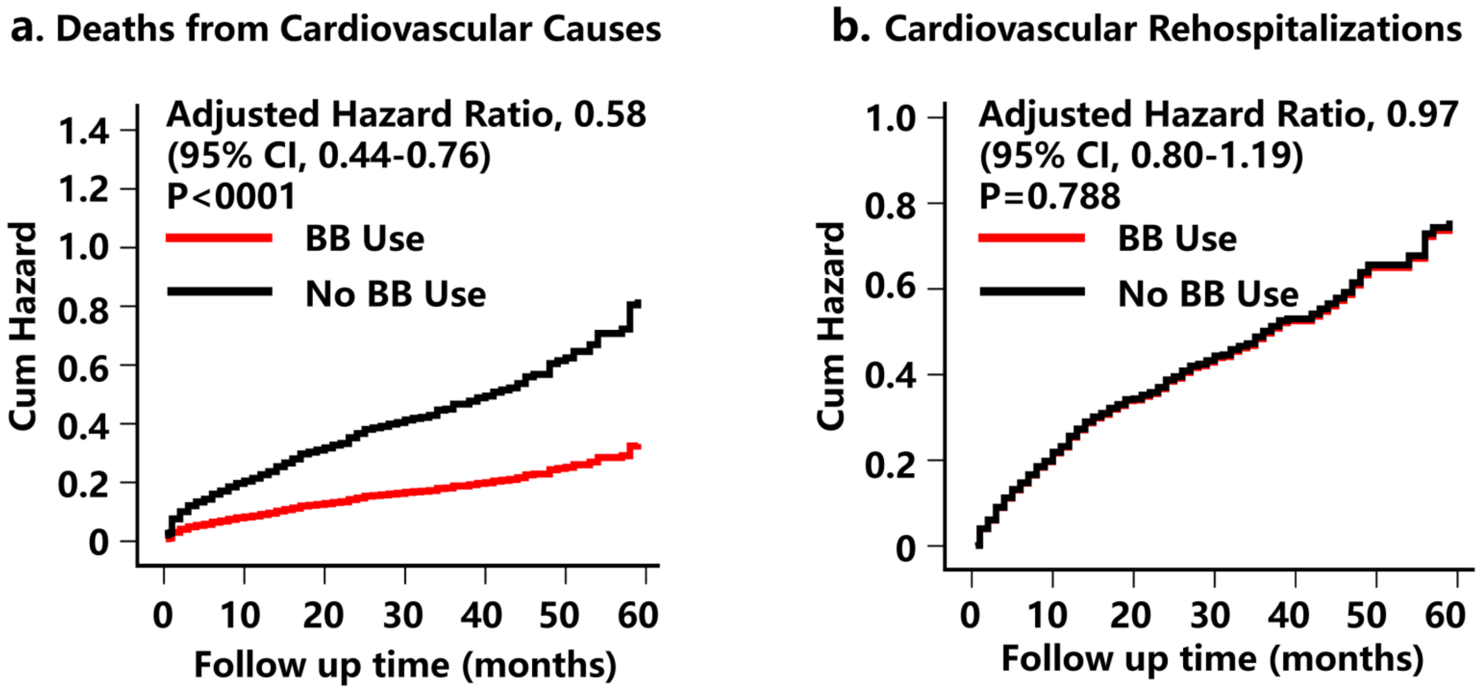
a. Deaths from Cardiovascular Causes
Adjusted Hazard Ratio, 0.58
(95% CI, 0.44-0.76)
P<0001
BB Use
No BB Use
Cum Hazard
1.4
1.2
1.0
0.8
0.6
0.4
0.2
0
0
10
20
30
40
50
60
Follow up time (months)
b. Cardiovascular Rehospitalizations
Adjusted Hazard Ratio, 0.97
(95% CI, 0.80-1.19)
P=0.788
BB Use
No BB Use
Cum Hazard
1.0
0.8
0.6
0.4
0.2
0
0
10
20
30
40
50
60
Follow up time (months)

Fig. S2. Effects of β-Blocker Treatment on Cardiovascular Deaths and Rehospitalization in Heart Failure

Among the entire cohort, β-blocker treatment (indicated in red) was significantly associated with a reduced risk of (a) the individual end point of death from a cardiovascular cause (adjusted HR, 0.58; 95% CI, 0.44 to 0.76; $P<0.001$), but not (b) the risk of rehospitalization owing to a cardiovascular cause (adjusted HR, 0.97; 95% CI, 0.80 to 1.19; $P=0.788$). Abbreviations: CI, confidence interval; HR, hazard ratio.

Fig. S3

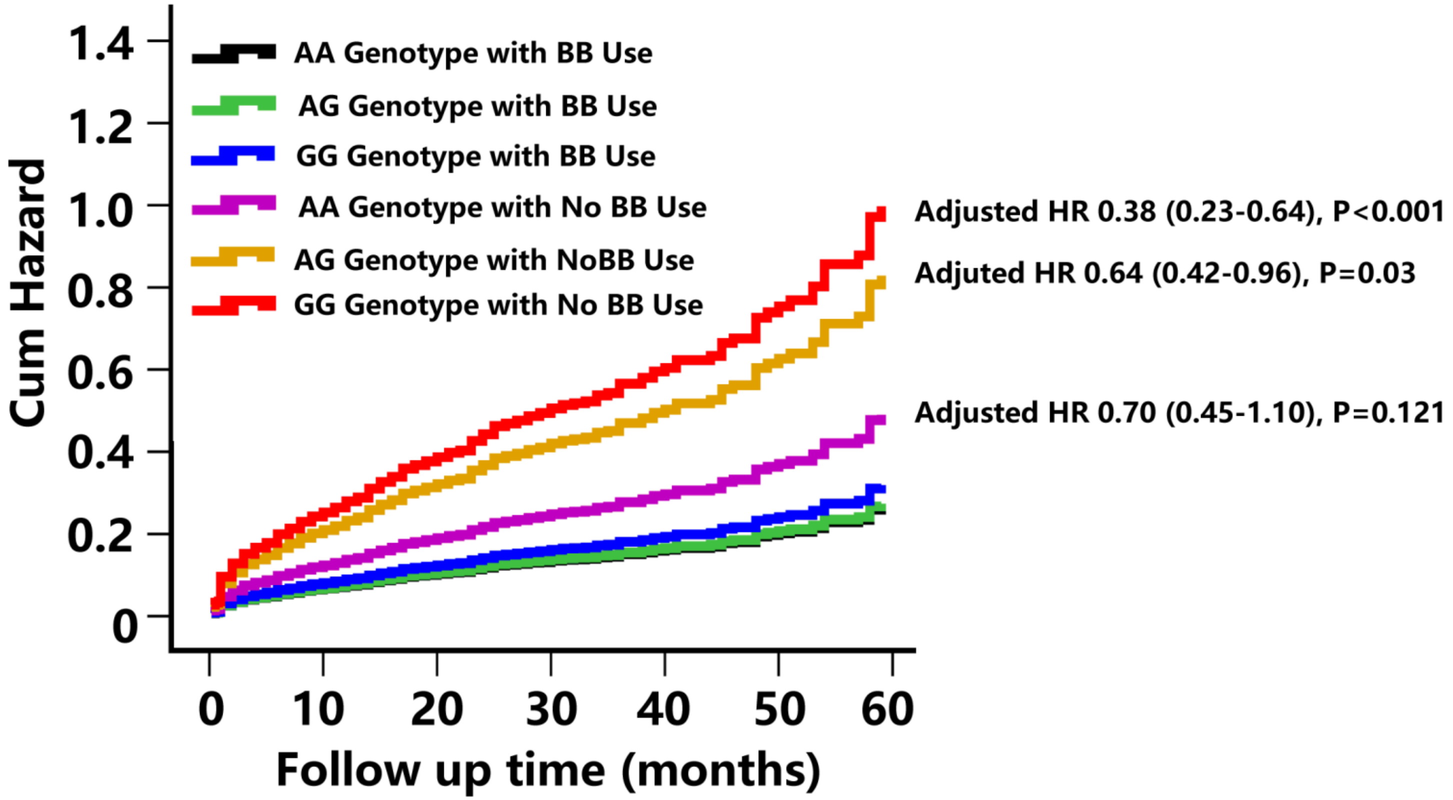
Cum Hazard
1.4
1.2
1.0
0.8
0.6
0.4
0.2
0
0
10
20
30
40
50
60
Follow up time (months)
AA Genotype with BB Use
AG Genotype with BB Use
GG Genotype with BB Use
AA Genotype with No BB Use
AG Genotype with NoBB Use
GG Genotype with No BB Use
Adjusted HR 0.38 (0.23-0.64), P<0.001
Adjuted HR 0.64 (0.42-0.96), P=0.03
Adjusted HR 0.70 (0.45-1.10), P=0.121

Fig. S3 Response to β-blocker of Patients with Different Genotype of *ADRB2* Arg16Gly

Carriers of the risk G allele (Gly16) were benefited from βAR-blocker treatment in an allele-dose-dependent manner. The cumulative hazard curves for the AA homozygotes with and without β-blocker treatment (BB) are in black and in purple, respectively; those for AG heterozygotes are in green and in orange, and those for the GG homozygotes are in blue and in red.

Fig. S4

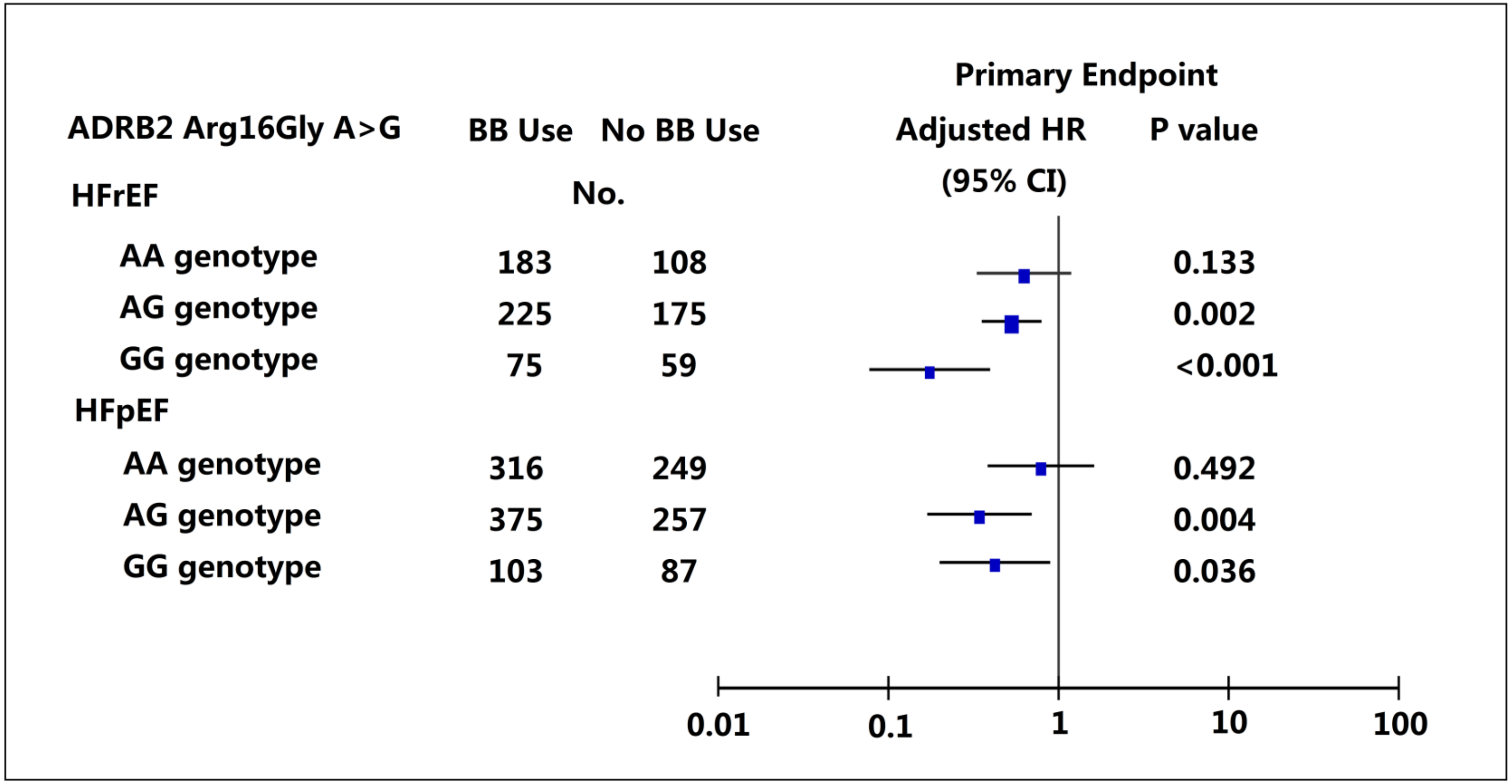
Primary Endpoint
ADRB2 Arg16Gly A>G
BB Use
No BB Use
Adjusted HR
(95% CI)
P value
HFrEF
No.
AA genotype
183
108
0.133
AG genotype
225
175
0.002
GG genotype
75
59
<0.001
HFpEF
AA genotype
316
249
0.492
AG genotype
375
257
0.004
GG genotype
103
87
0.036
0.01
0.1
1
10
100

Fig. S4. Effects of β-Blocker Treatment on the Primary Endpoint in the Subgroups Stratified by Left Ventricular Ejection Fraction

Patients who are homozygous for Arg16 genotype (AA genotype) of *ADRB2* Arg16Gly site showed no response to β-blockers no matter whether their ejection fractions were reduced or preserved (adjusted HR=0.62, 95%CI 0.33 to 1.16, P=0.133 and adjusted HR=0.78, 95% CI 0.38-1.59, P=0.492 respectively). However, Gly16 carriers (AG and GG genotype) were good responders to β-blockers no matter whether the patients had reduced (adjusted HR=0.54, 95% CI 0.36-0.80, P=0.002 for AG genotype and adjusted HR=0.17, 95% CI 0.08-0.40,P<0.001 for GG genotype) or preserved (adjusted HR=0.36, 95% CI 0.18-0.72, P=0.004 and adjusted HR=0.46, 95% CI 0.22-0.95, P=0.036) ejection fractions.

Fig. S5

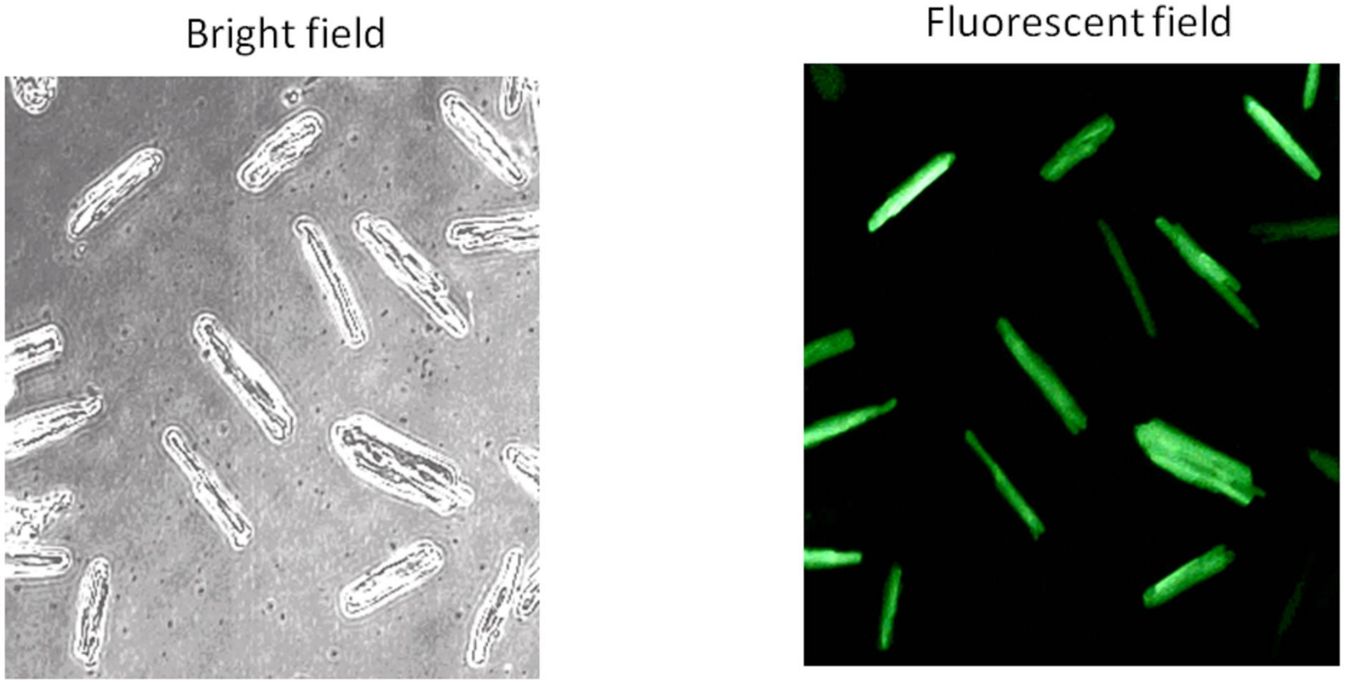

Fig. S5. Transduction Efficiency of the Adeno-GFP by Means of Fluorescent Microscopy.

A transduction efficiency of more than 90% was obtained with Adeno-GFP at 100 multiplicity of infection (moi) in rat cardiac myocytes.

The transduction method had a low toxicity since more than 90% of the transduced cells maintained a healthy rod-shape morphology.

Fig. S6

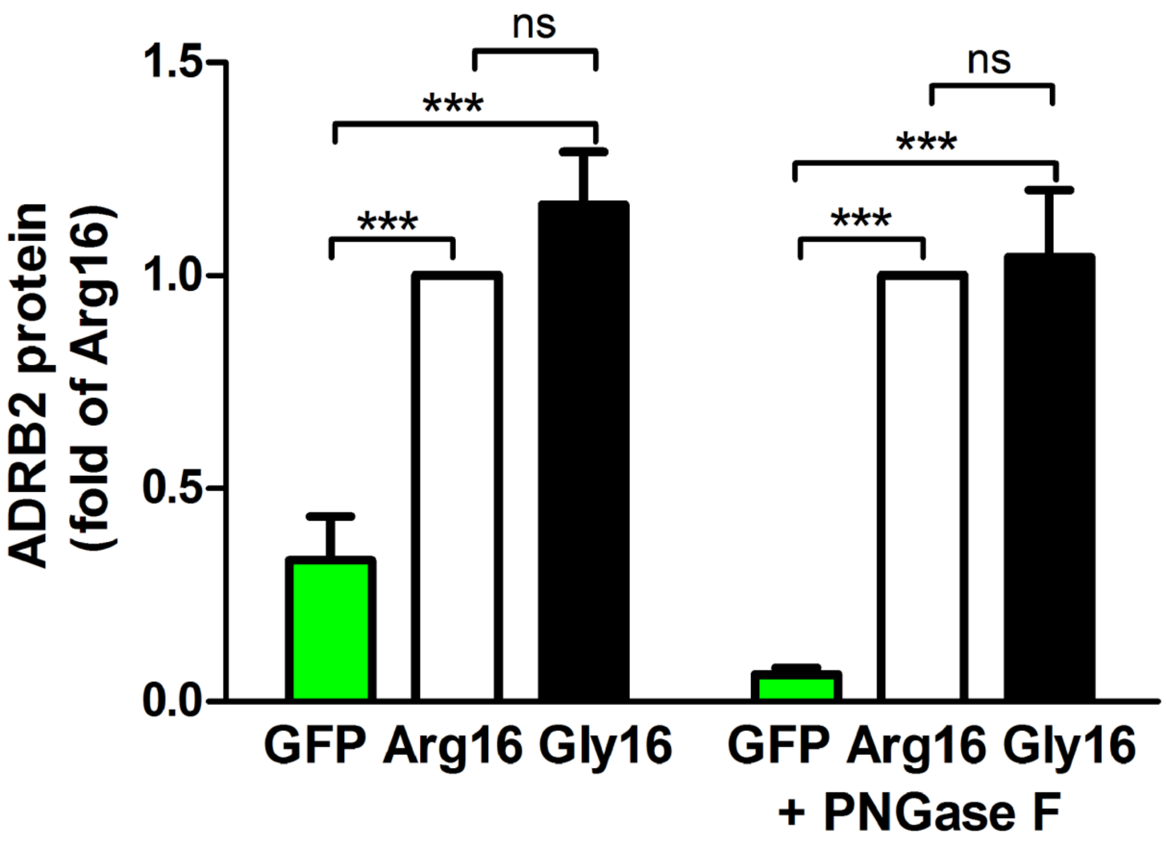

ns
***
***
ns
***
***
1.5
1.0
0.5
0.0
ADRB2 protein
(fold of Arg16)
GFP Arg16 Gly16
GFP Arg16 Gly16
+ PNGase F

Fig. S6. Expression Levels of β2AR with Different Genotypes at the *ADRB2* Arg16Gly Locus

The expression of β2AR in rat cardiac myocytes infected with 100 moi of adenoviruses encoding for GFP, *ADRB2*-Arg16 or *ADRB2*-Gly16 was detected by Western blotting method. Averaged data of band intensities are shown. Expression levels are presented as fold change of the *ADRB2*-Arg16 group (Means±SEM, N=7-10). No differences were found (by unpaired Student's *t*-test) between the *ADRB2*-Arg16 group and the *ADRB2*-Gly16 group for samples with and without deglycosylation treatment with PNGase F. ***: $P<0.001$, ns: $P>0.05$.

Fig. S7

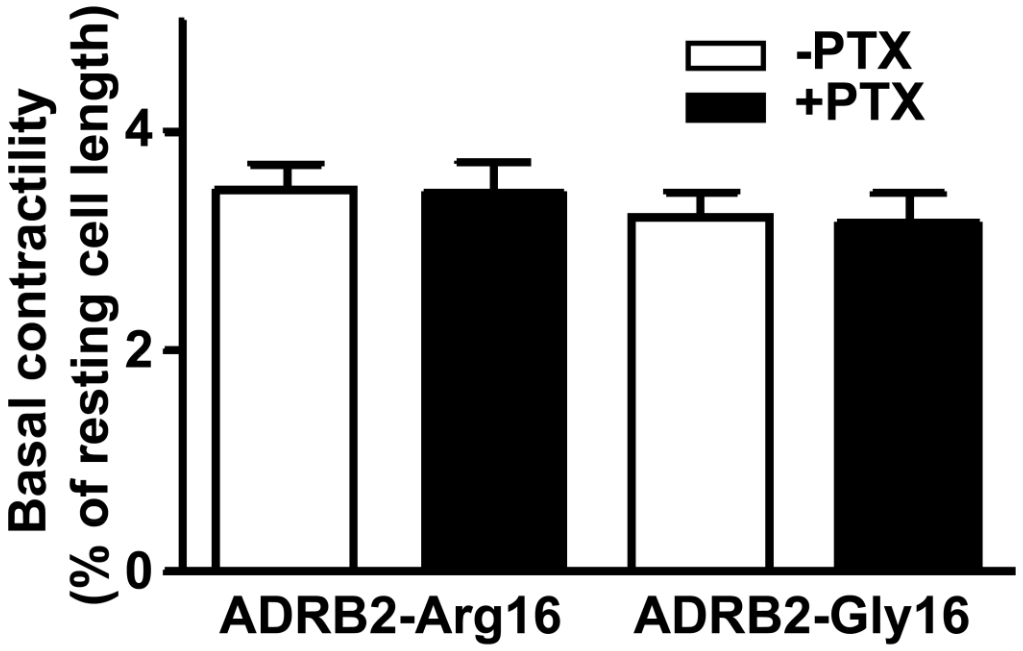
-PTX
+PTX
Basal contractility
(% of resting cell length)
4
2
0
ADRB2-Arg16
ADRB2-Gly16

Fig. S7. Basal Contractilities of Cultured ADRB2 Knockout Mouse Cardiomyocytes after Adenoviral Gene Transfer of ADRB2-Arg16 or ADRB2-Gly16 and Treatment with Pertussis Toxin (PTX). Data are Means±SEM (N=30-41 cells from 4 mice for each data point).
